# Z-α_1_-antitrypsin polymers impose molecular filtration in the endoplasmic reticulum after undergoing phase transition to a solid state

**DOI:** 10.1126/sciadv.abm2094

**Published:** 2022-04-08

**Authors:** Joseph E. Chambers, Nikita Zubkov, Markéta Kubánková, Jonathon Nixon-Abell, Ioanna Mela, Susana Abreu, Max Schwiening, Giulia Lavarda, Ismael López-Duarte, Jennifer A. Dickens, Tomás Torres, Clemens F. Kaminski, Liam J. Holt, Edward Avezov, James A. Huntington, Peter St George-Hyslop, Marina K. Kuimova, Stefan J. Marciniak

**Affiliations:** 1 Cambridge Institute for Medical Research (CIMR), Department of Medicine, University of Cambridge, The Keith Peters Building, Hills Road, Cambridge CB2 0XY, UK.; 2 Department of Chemistry, Imperial College London, Wood Lane, London W12 0BZ, UK.; 3 Department of Chemical Engineering and Biotechnology, University of Cambridge, Philippa Fawcett Drive, Cambridge CB3 0AS, UK.; 4 Departamento de Química Orgánica and Institute for Advanced Research in Chemical Sciences (IAdChem), Universidad Autónoma de Madrid, Madrid 28049, Spain.; 5 IMDEA Nanociencia, Campus de Cantoblanco, Madrid 28049, Spain.; 6 Institute for Systems Genetics, New York University Grossman School of Medicine, 435 E 30th St, New York, NY 10016, USA.; 7 Department of Clinical Neurosciences and UK Dementia Research Institute, University of Cambridge, Cambridge CB2 0AH, UK.; 8 Department of Medicine (Neurology), Temerty Faculty of Medicine, University of Toronto, University Health Network, Toronto, ON M5T 0S8, Canada.; 9 Taub Institute For Research on Alzheimer’s Disease and the Ageing Brain, Department of Neurology, Columbia University Irvine Medical Center, 630 West 1/68 Street, New York, NY 10032, USA.; 10 Royal Papworth Hospital, Cambridge CB2 0AY, UK.

## Abstract

Misfolding of secretory proteins in the endoplasmic reticulum (ER) features in many human diseases. In α_1_-antitrypsin deficiency, the pathogenic Z variant aberrantly assembles into polymers in the hepatocyte ER, leading to cirrhosis. We show that α_1_-antitrypsin polymers undergo a liquid:solid phase transition, forming a protein matrix that retards mobility of ER proteins by size-dependent molecular filtration. The Z-α_1_-antitrypsin phase transition is promoted during ER stress by an ATF6-mediated unfolded protein response. Furthermore, the ER chaperone calreticulin promotes Z-α_1_-antitrypsin solidification and increases protein matrix stiffness. Single-particle tracking reveals that solidification initiates in cells with normal ER morphology, previously assumed to represent a healthy pool. We show that Z-α_1_-antitrypsin–induced hypersensitivity to ER stress can be explained by immobilization of ER chaperones within the polymer matrix. This previously unidentified mechanism of ER dysfunction provides a template for understanding a diverse group of related proteinopathies and identifies ER chaperones as potential therapeutic targets.

## Teaser

Z-α_1_-antitrypsin polymers undergo phase transition to a solid matrix that immobilizes proteins in the endoplasmic reticulum.

## INTRODUCTION

A wide variety of conformational disorders arises when protein folding is compromised by gene mutation, aberrant posttranslational modification, or imbalances in the stoichiometry of protein complexes (*1*, *2*). α_1_-antitrypsin deficiency is an archetypal serine protease inhibitor (serpin) conformational disorder that results from mutations in the *SERPINA1* gene and is associated with both loss-of-function toxicity and toxic gain of function (*3*). α_1_-antitrypsin is produced predominantly by hepatocytes and acts to control inflammation and matrix degradation in distant tissues, most notably in the lungs, by inhibiting proteases released by neutrophils (*4*). The most common E342K pathogenic variant of α_1_-antitrypsin is the Z allele (Z-α_1_-antitrypsin). Wild-type M-α_1_-antitrypsin is not thought to form higher-order assemblies, but the Z-α_1_-antitrypsin mutant allele generates a protein that can form ordered linear polymers (*5*). Polymerization occurs in the lumen of the endoplasmic reticulum (ER) where proteins destined for the cell surface or secretion fold to acquire their native structure. Accumulation of Z-α_1_-antitrypsin polymers is accompanied by fragmentation of the ER into large vesicular structures referred to as ER inclusions. ER inclusions are associated with toxic gain of function, which causes liver cirrhosis that accounts for 10% of deaths in individuals homozygous for the Z allele (Pi*ZZ) (*6*–*8*). In addition, the Z allele is overrepresented in patients requiring liver transplantation for both nonalcoholic steatohepatitis and alcoholic liver cirrhosis (*9*), indicating its involvement in common diseases.

We hypothesized that accumulation of polymerized α_1_-antitrypsin could alter ER function by changing the biophysical properties of the protein milieu within the lumen. To investigate this, we characterized the mobility of α_1_-antitrypsin and other proteins in the ER of live cells and found that Z-α_1_-antitrypsin can form a solid matrix of protein polymers that fills ER inclusions and impedes the mobility of other ER proteins, acting as a size-dependent molecular sieve. Furthermore, we provide evidence that calreticulin, an abundant lectin chaperone that interacts with α_1_-antitrypsin during folding, promotes the formation of solid-phase Z-α_1_-antitrypsin, increasing Z-α_1_-antitrypsin polymer length and, in turn, the stiffness of the protein matrix, which immobilizes the chaperone. Solidification of Z-α_1_-antitrypsin was also promoted by ER stress–induced activation of the unfolded protein response (UPR), a collection of pathways that defend cells against partially folded and misfolded ER proteins (*10*). Z-α_1_-antitrypsin solidification during ER stress was ameliorated by inhibition of the activating transcription factor 6 (ATF6) branch of the UPR, which up-regulates calreticulin. Notably, the UPR is not activated by the accumulation of Z-α_1_-antitrypsin polymers alone (*11*–*14*), but cells bearing the Z-α_1_-antitrypsin allele are hypersensitive to orthogonal stresses that induce the UPR. The mechanism for this hypersensitivity has remained mysterious but has been suggested as a cause for hepatotoxicity in α_1_-antitrypsin deficiency (*12*, *13*). We propose that the bidirectional relationship between UPR-induced chaperones and the solid state of Z-α_1_-antitrypsin explains the heightened sensitivity of Z-α_1_-antitrypsin–expressing cells to ER stress. The filtration of ER proteins imposed by solid-phase Z-α_1_-antitrypsin provides rare insight into the consequences of protein phase transitions within living cells, offering additional mechanistic understanding of the pathology of proteinopathies, and suggests new therapeutic targets.

## RESULTS

### Z-α_1_-antitrypsin accumulation alters biophysical properties within ER inclusions

**Fig. 1.**
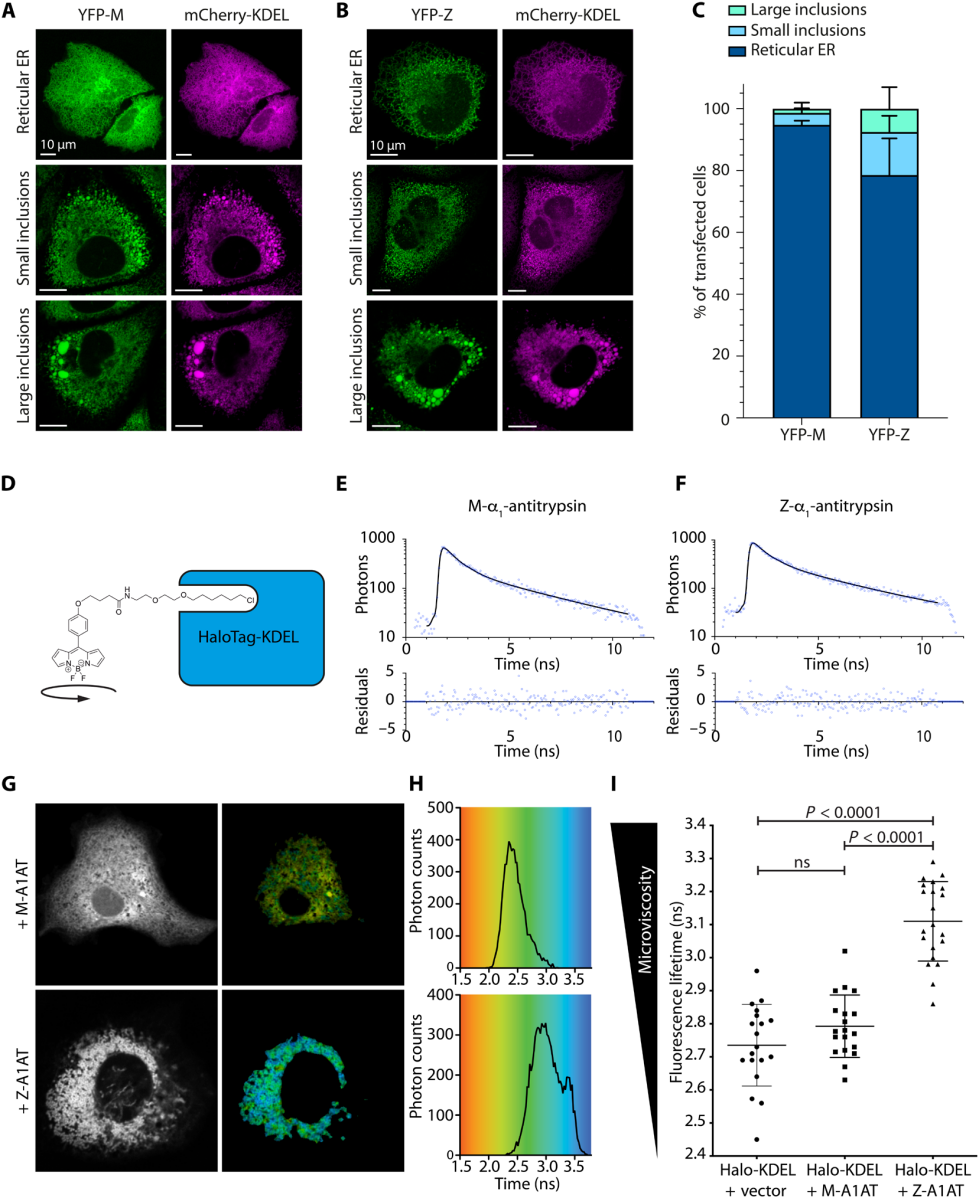
Z-α_1_-antitrypsin expression fragments the ER and increases microviscosity in the ER lumen. Reticular, small-inclusion, and large-inclusion ER morphologies in CHO-K1 cells coexpressing mCherry-KDEL with (**A**) YFP-tagged M-α_1_-antitrypsin (YFP-M) or (**B**) YFP-Z. Intensity gamma manipulation of 0.5 was applied to enable visualization of both reticular ER and ER inclusions. (**C**) Proportion of transfected cells displaying ER morphologies. (**D**) Chemical structure of microviscosity-sensitive BODIPY-HaloLigand cartooned with HaloTag protein. (**E** and **F**) Example fluorescence decay curves (top) and residuals (bottom) of BODIPY-HaloTag ROVI probe localized to the ER of CHO-K1 cells expressing (E) M-α_1_-antitrypsin or (F) Z-α_1_-antitrypsin. (**G**) Fluorescence intensity images (left) and color-coded fluorescence lifetime images (right). (**H**) Fluorescence lifetime distribution histogram of CHO-K1 cells expressing HaloTag-KDEL labeled with BODIPY-HaloLigand with either M-α_1_-antitrypsin (top) or Z-α_1_-antitrypsin (bottom). (**I**) Mean ER fluorescence lifetime (corresponding to microviscosity) of CHO-K1 cells transiently transfected with HaloTag-KDEL together with either an empty vector control (Vector), M-α_1_-antitrypsin (M-A1AT), or Z-α_1_-antitrypsin (Z-A1AT) before labeling with BODIPY-HaloLigand and ER lumenal microviscosity quantitation by FLIM. Statistical significance was analyzed by analysis of variance (ANOVA) with Bonferroni’s multiple comparison test. A minimum of 19 cells were analyzed over three independent repeats. ns, not significant.

Cells expressing Z-α_1_-antitrypsin display a range of ER morphologies, from a normal reticular ER to complete ER fragmentation (*15*). We previously established a CHO-K1 cell model expressing α_1_-antitrypsin fused to fluorescent proteins to facilitate imaging studies (*16*). We further validated the CHO-K1 model by comparison with our published hepatocyte-like cells (HLCs) derived from induced pluripotent stem cells (iPSCs) (*15*). CHO-K1 cells made to express yellow fluorescent protein (YFP)–tagged Z-α_1_-antitrypsin (YFP-Z) displayed a spectrum of ER morphologies, faithfully reproducing those observed in Pi*ZZ iPSC-derived HLCs with similar frequency (Fig. 1, A to C) (*15*).

A major function of the ER is to support protein folding, which is dependent on molecular motion. The rate of movement of amino acid side chains during protein folding is strongly influenced by environmental microviscosity (*17*). At another level, translational motion of proteins facilitates binding and release of molecular chaperones to nascent proteins to promote correct protein folding (*18*, *19*), which is governed both by microviscosity and crowding/confinement by other macromolecules (*20*). To investigate the effect of α_1_-antitrypsin accumulation on the biophysical properties of the contents of the ER, we assessed ER microviscosity in live cells expressing M- or Z-α_1_-antitrypsin using rotor-based organelle viscosity imaging (ROVI). A small cell-permeable fluorescent molecular rotor was covalently bound to a genetically encoded HaloTag that was targeted to the ER using a signal sequence (Fig. 1D) (*21*–*23*). Fluorescence lifetime of the rotor reports on local microviscosity (*23*). ER lumen–targeted ROVI was applied to CHO-K1 cells expressing either wild-type M-α_1_-antitrypsin or polymerogenic Z-α_1_-antitrypsin (Fig. 1, E to I). As previously reported, expression of Z-α_1_-antitrypsin caused variable fragmentation of the ER network (Fig. 1G, bottom) (*13*, *16*). Fluorescence lifetime imaging microscopy (FLIM) of the rotor revealed a significant increase in ER lumenal microviscosity in Z-α_1_-antitrypsin–expressing cells compared with cells expressing M- or no α_1_-antitrypsin (Fig. 1, G to I). This observation demonstrates that Z-α_1_-antitrypsin accumulation affects molecular motion in the ER lumen.

### Z-α_1_-antitrypsin reduces protein mobility within ER inclusions

**Fig. 2.**
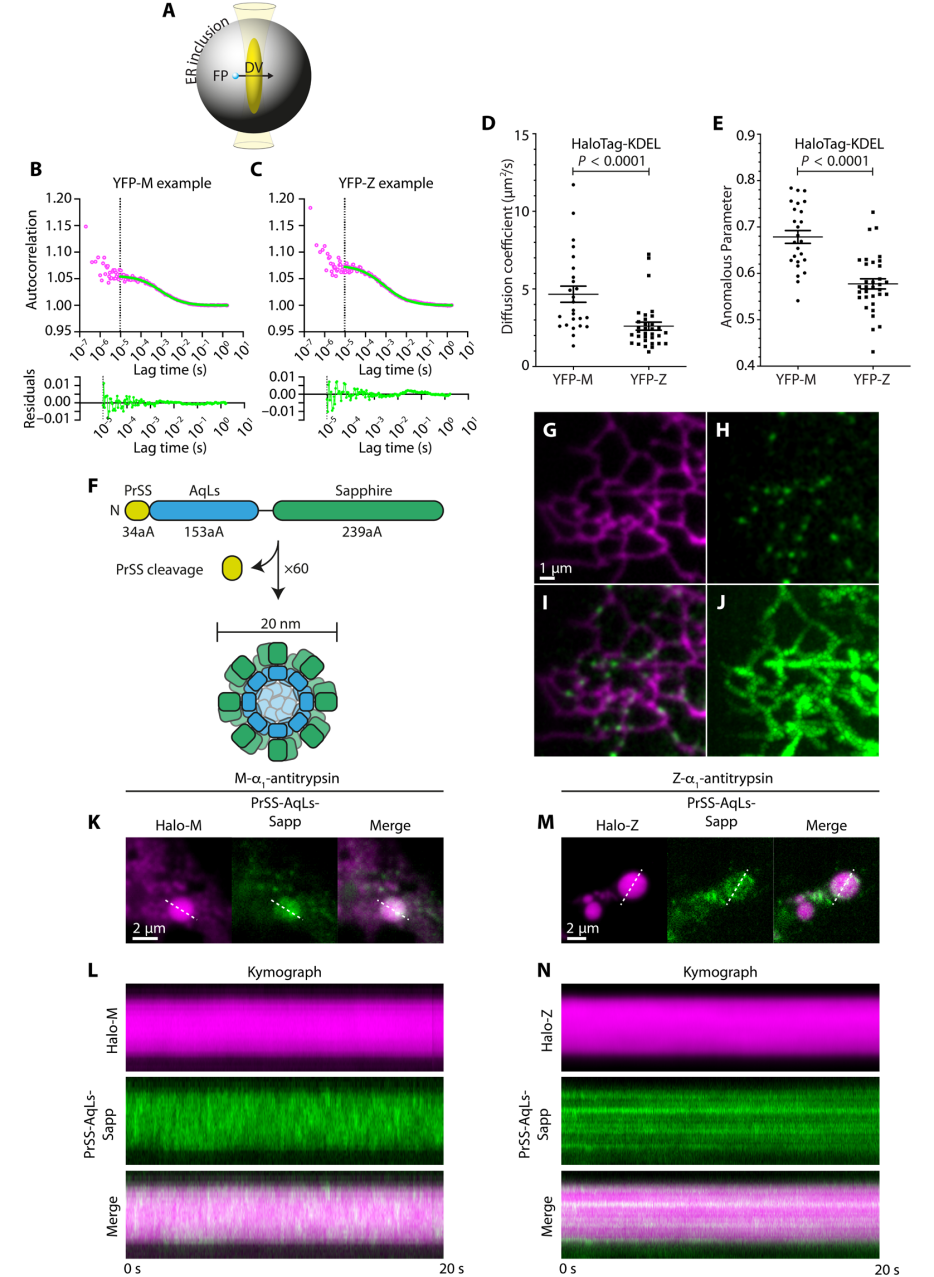
Reduced mobility of inert proteins in ER inclusions suggests molecular filtration imposed by Z-α_1_-antitrypsin. CHO-K1 cells were transfected with YFP-M or YFP-Z and a HaloTagged ER protein 48 hours before FCS in large ER inclusions. (**A**) Schematic showing FCS detection volume (DV) within an ER inclusion being traversed by a fluorescent particle (FP). (**B** and **C**) Example FCS autocorrelation curves (top) and residuals (bottom) for HaloTag-KDEL in cells expressing (B) YFP-M or (C) YFP-Z. (**D**) Measured effective diffusion coefficients (*D*_eff_) for HaloTag-KDEL and (**E**) the corresponding anomalous parameter of diffusion (α). A minimum of 25 cells were analyzed, acquired over three independent experiments. (**F**) Domain organization of ER-AqLs-Sapphire; preprolactin signal sequence (PrSS), the *Aquifex aeolicus* lumazine synthase scaffold (AqLs), and Sapphire fluorescent protein (aA denotes number of amino acids). (**G** to **J**) HILO micrographs of ER in a CHO-K1 cell coexpressing (G) Sec61TA-HaloTag (JF646 labeled) and (H) AqLs-Sapphire, merged in (I). (J) Maximum intensity projection of AqLs intensity from 1000 frames acquired over 20 s. (**K** to **N**) ER inclusion containing (K) HaloTagged–M-α_1_-antitrypsin (Halo-M) or (M) HaloTagged–Z-α_1_-antitrypsin (Halo-Z) (JF646 labeled) and AqLs-Sapphire. Dashed lines denote ROIs used to generate fluorescence kymographs [(L) and (N), respectively], displaying a 20-s imaging period. Images are representative of three independent repeats.

To test directly whether the changes in ER microviscosity are accompanied by altered protein mobility, we next used fluorescence correlation spectroscopy (FCS) to measure protein diffusion in the ER lumen. To circumvent confounding effects of complex ER network geometry (*24*), we limited our analyses to large ER inclusions (greater than 1 μm in diameter). FCS uses fluctuations in fluorescence intensity within femtoliter volumes to report on diffusion with millisecond resolution (*25*). To discern the effect of Z-α_1_-antitrypsin on small-protein mobility in ER inclusions, FCS was used to measure diffusion of the small inert protein HaloTag, localised to the ER lumen with a C-terminal KDEL motif (HaloTag-KDEL) in ER inclusions of YFP-Z and in rare inclusions of YFP-tagged M-α_1_-antitrypsin (YFP-M), producing autocorrelation curves with good fit (Fig. 2, A to C). HaloTag-KDEL displayed a lower effective diffusion coefficient (*D*_eff_) in inclusions of Z-α_1_-antitrypsin–expressing cells compared to those expressing M-α_1_-antitrypsin (2.6 ± 0.3 μm^2^/s versus 4.7 ± 0.5 μm^2^/s; Fig. 2D). For diffusion-dominated molecular motion, the mean-squared displacement <*R*^2^(*t*)> scales as a power law with time. The exponent α (denoting an anomalous diffusion parameter) can be used to broadly classify motion as “diffusive” (α = 1), “superdiffusive” (α > 1), or “subdiffusive” (α < 1) (derived in Materials and Methods) (*26*). Protein mobility in cellular environments can often display motion consistent with anomalous diffusion as a result of crowding or confinement (*27*, *28*). The reduction in *D*_eff_ of HaloTag-KDEL was accompanied by a significant reduction in α from 0.68 ± 0.01 in YFP-M–expressing cells to 0.58 ± 0.01 in YFP-Z–expressing cells (Fig. 2E), indicating increased molecular confinement in the presence of Z-α_1_-antitrypsin. In the context of the geometry of large ER inclusions (in both Z- and M-α_1_-antitrypsin–expressing cells), such confinement cannot be attributed to direct collision of proteins with the encapsulating ER membrane. Hence, these data suggested that Z-α_1_-antitrypsin itself might directly restrict and confine the mobility of small proteins in the ER lumen.

As many chaperones and folding factors that support ER protein folding assemble into higher-order complexes (*29*, *30*), we next assessed the effect of Z-α_1_-antitrypsin expression on mobility of larger protein assemblies. To this end, we generated an ER-localizing *Aquifex aeolicus* lumazine synthase scaffold (ER-AqLs), a spherical genetically encoded multimeric nanoparticle (GEM) with diameter of 20 nm (Fig. 2F) (*31*). ER-AqLs punctate particles could readily be observed to colocalize with an ER marker protein in cells imaged by highly inclined and laminated optical sheet (HILO) microscopy (Fig. 2, G to J). ER-AqLs was then expressed in CHO-K1 cells together with HaloTagged α_1_-antitrypsin (*16*) and imaged at a frame rate of 50 Hz. Kymographs display ER-AqLs intensity fluctuations over time through a linear region of interest (ROI) bisecting the inclusion (Fig. 2, K to N). In inclusions of cells expressing HaloTag–M-α_1_-antitrypsin, mobile ER-AqLs GEMs could be visualized within ER inclusions [Fig. 2, K and L, and movie S1 (top)]. In some cells expressing HaloTag–Z-α_1_-antitrypsin, ER-AqLs displayed relatively homogeneous distribution of fluorescence (fig. S1), consistent with a rapid GEM diffusion or high GEM density preventing particle resolution. However, a subpopulation of HaloTag–Z-α_1_-antitrypsin–expressing cells displayed ER-AqLs puncta that remained static throughout the course of the experiment [Fig. 2, M and N, and movie S1 (bottom)]. This suggests that large protein complexes can become immobilized by physical confinement in Z-α_1_-antitrypsin inclusions. Together, these FCS and HILO data indicate that Z-α_1_-antitrypsin reduces the mobility of both small proteins and large protein assemblies within ER inclusions. The different degrees to which these inert protein species of differing sizes are immobilized suggested a model whereby Z-α_1_-antitrypsin imposes size-dependent molecular filtration on the contents of ER inclusions.

### Z-α_1_-antitrypsin adopts a low mobility state in the ER

**Fig. 3.**
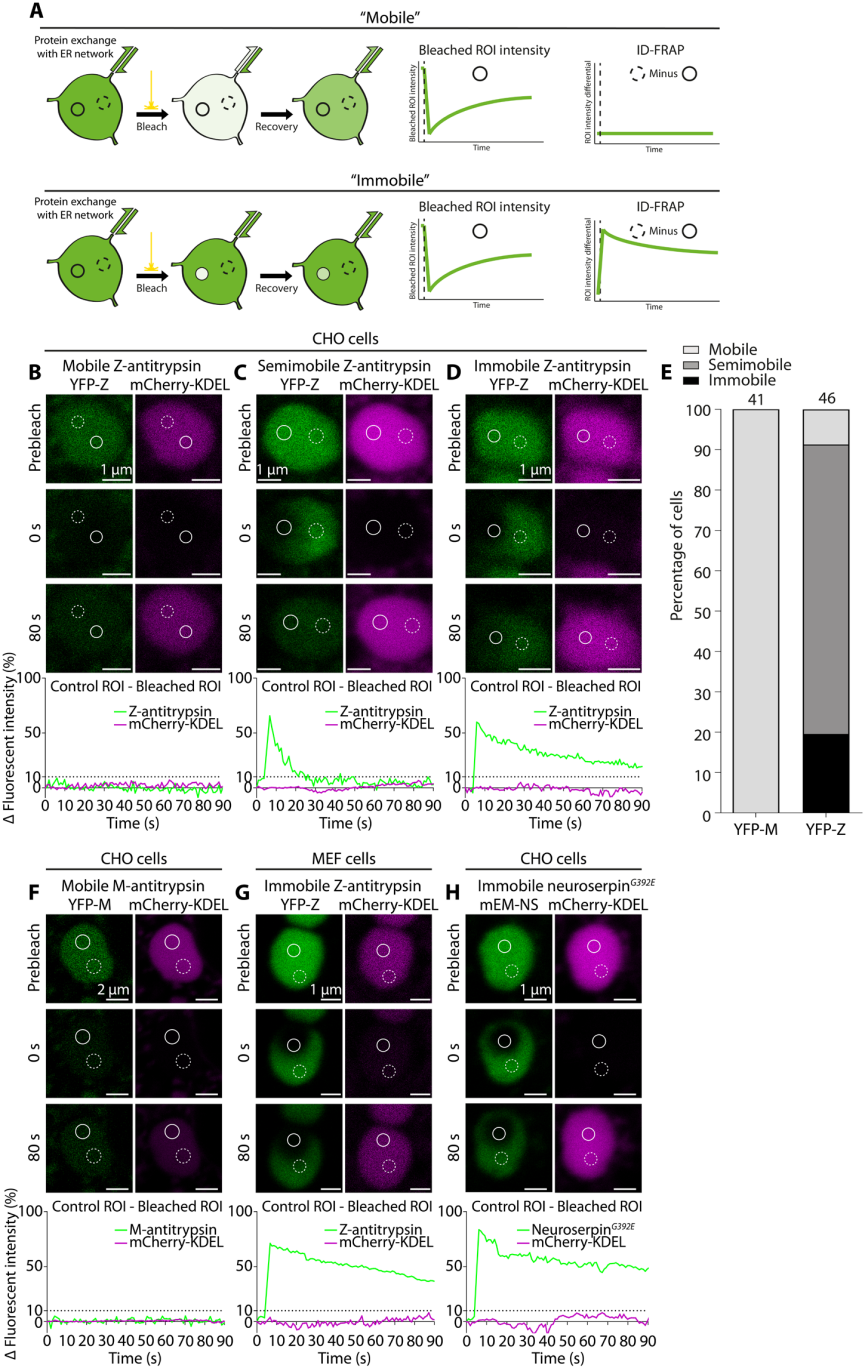
Z-α_1_-antitrypsin adopts a spectrum of mobilities in ER inclusions. (**A**) Schematic depicting photobleaching of fluorescent protein (green) within an ER inclusion that can exchange fluorescent protein with the ER network. Solid circle represents a bleached ROI, while the dashed-line circle represents a control ROI. Represented are two situations, showing a fluorescent protein with relatively high (top) or low (bottom) mobility. Cartooned graphs show the bleached ROI intensity (left) versus control ROI intensity minus bleached ROI intensity (right) as used in intensity differential (Δ*I*) FRAP (ID-FRAP). (**B** to **D**) Representative examples of CHO-K1 cell large ER inclusions containing (B) mobile, (C) semi-mobile, and (D) immobile YFP-Z before and at 0 or 80 s after bleach. Δ*I* (Intensity^control^ − Intensity^bleached^) is shown as a percentage of the initial fluorescence intensity. (**E**) Distribution of YFP-M or YFP-Z mobilities (coexpressed with mCherry-KDEL) in CHO-K1 cell large ER inclusions determined by ID-FRAP, shown as a percentage of cells analyzed. (**F**) A representative example of large ER inclusions containing mobile M-α_1_-antitrypsin. (**G**) A representative example of large ER inclusions containing immobile YFP-Z coexpressed with mCherry-KDEL in a MEF. (**H**) A representative example of a large ER inclusion containing immobile mEmerald-tagged neuroserpin*^G392E^* in a CHO-K1 cell, coexpressed with mCherry-KDEL.

We previously reported that YFP-Z recovers slowly from photobleaching within ER inclusions (*16*). This, combined with the effects of Z-α_1_-antitrypsin on ER microviscosity and protein mobility, led us to explore the mobility of Z-α_1_-antitrypsin in large ER inclusions. The application of fluorescence recovery after photobleaching (FRAP) to assess α_1_-antitrypsin mobility within individual ER inclusions is hampered by variable connectivity between inclusions and the rest of the ER network (Fig. 3A, “Bleached ROI intensity” graphs) (*16*). To overcome this problem, we implemented a modified FRAP protocol, referred to here as intensity differential FRAP (ID-FRAP), to report on intrainclusion protein mobility with minimal confounding influence from ER connectivity. ID-FRAP used the intensity differential (Δ*I*) between two ROIs within a single inclusion, bleached versus unbleached control (Fig. 3A, “ID-FRAP” graphs). Two-color ID-FRAP was used to report mobility of YFP-Z and a small fluorescent ER marker mCherry-KDEL simultaneously, and ER inclusions were categorized into phenotypic groups on the basis of the Δ*I* of YFP-Z. Those in which Δ*I* failed to exceed 10% were described as inclusions with “mobile” contents (Fig. 3B); those in which Δ*I* recovered to below 10% within 80 s were described as “semi-mobile” (Fig. 3C), while those in which Δ*I* remained greater than 10% after 80 s were described as inclusions with “immobile” contents (Fig. 3D). The spread of YFP-Z mobilities in large inclusions of CHO cells showed that 19.6% of cells displayed immobile YFP-Z, 71.7% displayed semi-mobile YFP-Z, and 8.7% displayed mobile YFP-Z (Fig. 3E). In contrast, CHO cells expressing YFP-M all displayed a mobile phenotype (Fig. 3, E and F). As in CHO cells, immobile Z-α_1_-antitrypsin was readily seen in mouse embryonic fibroblasts (MEFs) transfected with YFP-Z (Fig. 3G).

We next addressed whether immobilization is a specific feature of Z-α_1_-antitrypsin or a generalizable feature of polymerogenic protein accumulation in the ER. Neuroserpin is a serpin family member expressed predominantly in the brain. Mutations in neuroserpin lead to polymerization and accumulation of the protein in the neuronal ER, causing the inherited dementia familial encephalopathy with neuroserpin inclusion bodies (FENIB) (*32*). We observed that mEmerald-tagged neuroserpin*^G392E^*, a mutant associated with early-onset FENIB, could form large ER inclusions with an immobile ID-FRAP phenotype in CHO cells (Fig. 3H).

Combined with the observation that both small ER proteins and large protein assemblies experience increased confinement within ER inclusions of YFP-Z (Fig. 2), these data indicate that Z-α_1_-antitrypsin mobility governs the mobility of other proteins in the ER. Moreover, this phenomenon likely represents a shared feature of polymerogenic serpin mutants associated with clinical features as diverse as cirrhosis and dementia.

### Z-α_1_-antitrypsin immobilization is promoted by the UPR

**Fig. 4.**
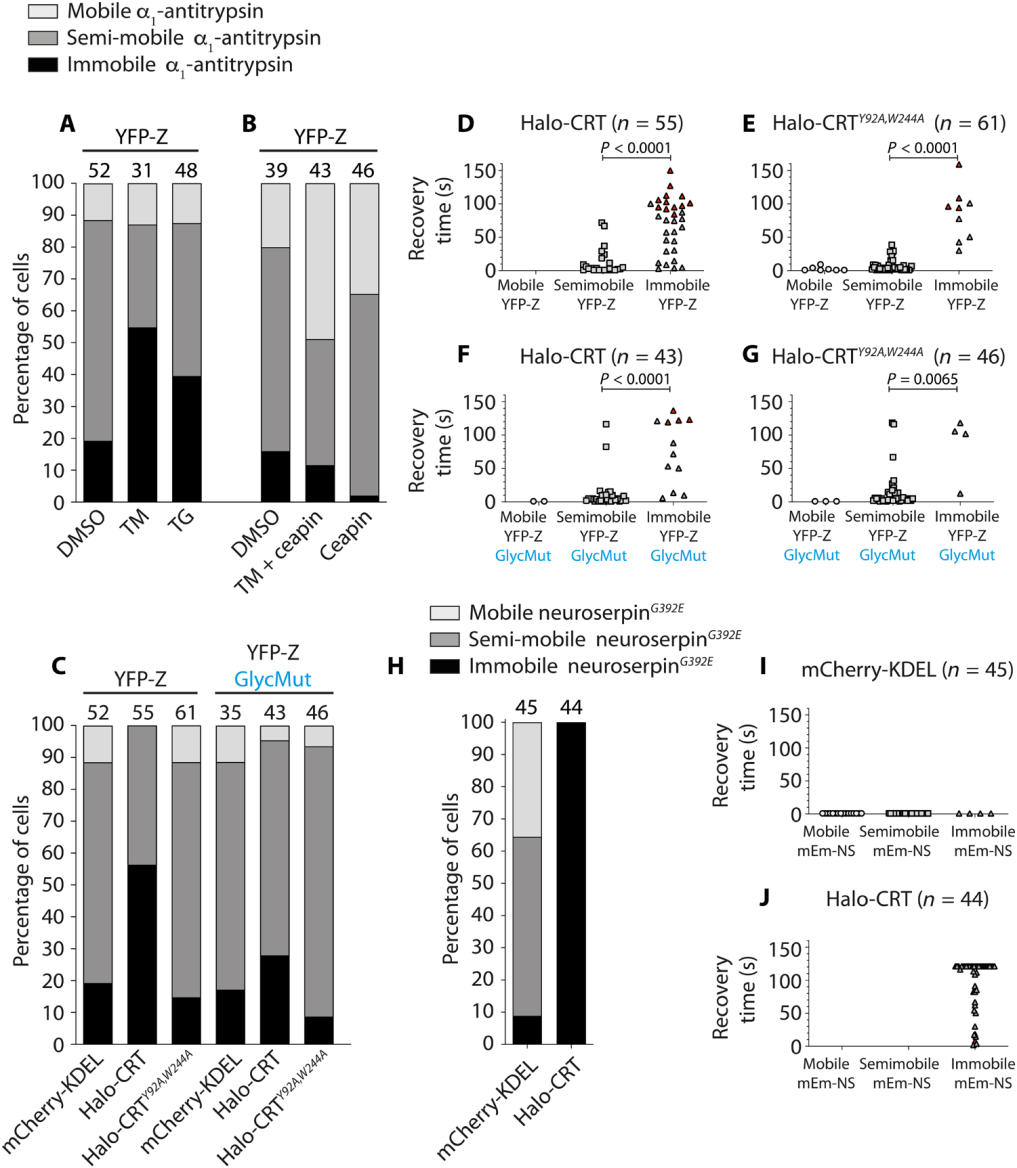
UPR activation and calreticulin overexpression reduces protein mobility in ER inclusions. (**A**) CHO-K1 cells were transfected with YFP-Z and ER-mCherry for 48 hours before treatment for 8 hours with either dimethyl sulfoxide (DMSO), 2 μg/ml tunicamycin (TM), or 0.02 μM thapsigargin (TG) before YFP-Z mobility assessment by ID-FRAP. The distribution of YFP-Z mobility phenotypes (mobile, semi-mobile, and immobile) was calculated as a percentage of cells analyzed. (**B**) CHO-K1 cells analyzed as in “A,” after treatment with 5 μM Ceapin A7 in combination with tunicamycin (2 μg/ml) or 5 μM Ceapin A7 alone. (**C**) Frequency distribution of YFP-Z or YFP-Z*^N46A,N83A,N247A^* (GlycMut) mobility phenotypes in CHO-K1 cells cotransfected with mCherry-KDEL, HaloTag-calreticulin (Halo-CRT), and HaloTag-calreticulin*^Y92A,W244A^* (Halo-CRT*^Y92A,W244A^*). The number of cells analyzed in (A) to (C) is noted above chart bars, acquired over a minimum of three independent experiments. (**D** to **G**) ID-FRAP recovery times of calreticulin variants from cells analyzed in (C), grouped by mobility phenotype of YFP-Z in that inclusion. *n* denotes the number of cells analyzed. Red points denote cells where homogenization had not been achieved by the end point of the experiment. *P* values were generated by Student’s *t* test. (**H**) CHO-K1 cells transfected with mEmerald-neuroserpin*^G392E^* and mCherry-KDEL or HaloTag-CRT for 48 hours were assessed by ID-FRAP to determine mEmerald-neuroserpin*^G392E^* mobility phenotype. ID-FRAP recovery times of (**I**) mCherry-KDEL or (**J**) Halo-CRT corresponding to cells in (H) are shown.

The reported hypersensitivity of Z-α_1_-antitrypsin–expressing cells to ER stress (*12*, *13*) led us to question whether an imbalance in ER proteostasis might contribute to formation of the immobile Z-α_1_-antitrypsin. Accordingly, CHO-K1 cells expressing YFP-Z and mCherry-KDEL for 48 hours were exposed to ER stress for a further 8 hours, either by treatment with tunicamycin, which inhibits N-linked glycosylation, or thapsigargin, which depletes ER calcium. Neither tunicamycin nor thapsigargin had a detectable effect on mobility of the inert ER marker mCherry-KDEL as reported by ID-FRAP recovery time (fig. S2), but both agents markedly increased the proportion of cells with immobile YFP-Z from 19.2 to 54.8% with tunicamycin and 39.6% with thapsigargin (Fig. 4A).

ER stress is accompanied by UPR activation that increases ER protein folding capacity, in part, through up-regulation of molecular chaperones (*10*). There are three canonical branches of the UPR, and the ATF6 branch drives a large proportion of UPR-induced chaperone up-regulation (*33*, *34*). In an attempt to uncouple the driver of ER stress (protein misfolding) from its downstream effects (via UPR activation), cells expressing YFP-Z were treated with tunicamycin in the presence of Ceapin A7, which inhibits the ATF6 branch of the UPR (*35*). ATF6 inhibition abolished the YFP-Z immobilizing effects of tunicamycin treatment (Fig. 4B), suggesting that induction of chaperones by the endogenous stress response may drive Z-α_1_-antitrypsin into the immobile state. Moreover, inhibition of basal ATF6 activity in the absence of ER stress decreased the basal proportion of cells displaying immobile YFP-Z and increased the proportion with the mobile phenotype (Fig. 4B). These data implicate ATF6-induced factors in the formation of a low mobility state of Z-α_1_-antitrypsin.

### Calreticulin drives the immobilization of Z-α_1_-antitrypsin

Calreticulin is a highly abundant soluble ER lectin chaperone that interacts with client proteins via N-linked glycans and is up-regulated by ATF6 (*34*). It is known to engage Z-α_1_-antitrypsin (*36*), which is glycosylated at three sites (*37*, *38*). Knockdown of calreticulin has been shown to decrease intracellular levels of Z-α_1_-antitrypsin by promoting its secretion (*39*). Hence, we postulated that calreticulin is a likely contributor to Z-α_1_-antitrypsin immobilization. Using two-color ID-FRAP, we assessed the mobility of Z-α_1_-antitrypsin (YFP-Z) coexpressed with HaloTagged calreticulin (HaloTag-CRT) in ER inclusions. Expression of wild-type HaloTag-CRT increased the proportion of cells with immobile YFP-Z from 17 to 55% (Fig. 4C). By contrast, expression of the HaloTag-CRT*^Y92A,W244A^* mutant, which binds neither substrate glycans nor the calreticulin-cofactor ERp57 (*40*, *41*), failed to drive YFP-Z immobilization (Fig. 4C). Similarly, neither wild-type HaloTag-CRT nor HaloTag-CRT*^Y92A,W244A^* affected the mobility of glycosylation-incompetent YFP-Z*^N46A,N83A,N247A^* (Fig. 4C). These results implicate calreticulin as an ATF6-transcriptional target that promotes Z-α_1_-antitrypsin immobilization.

In addition to assessing YFP-Z mobility, ID-FRAP recovery times [time taken for fluorescence intensity differential (Δ*I*) of a protein between control and bleach ROIs within the same inclusion to fall below 10%] were measured for coexpressed fluorescent proteins. Once again, YFP-Z had no effect on the small fluorescent protein mCherry-KDEL, which homogenized within 1 s after bleach (fig. S3A). By contrast, homogenization of HaloTag-CRT was notably slower in inclusions containing immobile YFP-Z (Fig. 4D). Although expression of HaloTag-CRT*^Y92A,W244A^* did not promote the immobile state of YFP-Z (Fig. 4C), the mobility of this inactive calreticulin mutant was reduced in cells with immobile YFP-Z to a similar extent as had been seen for wild-type HaloTag-CRT (Fig. 4D versus Fig. 4E). These effects on ER protein mobility did not require Z-α_1_-antitrypsin glycosylation, as similar values were observed in cells expressing nonglycosylated YFP-Z*^N46A,N83A,N247A^* (Fig. 4, F and G, and fig. S3B). Together, these data suggested that the reduction in the mobility of calreticulin in the presence of immobile YFP-Z was not likely to represent a chaperone-client interaction but was more likely the result of chaperone confinement by immobile YFP-Z.

To ensure that the fluorescent protein tag on Z-α_1_-antitrypsin did not contribute to reduced calreticulin mobility, a single-color ID-FRAP experiment was performed in a CHO-K1 stable cell line expressing untagged Z-α_1_-antitrypsin under a dox-inducible promoter (*13*). Recovery time distributions for coexpressed HaloTag-CRT and HaloTag-CRT*^Y92A,W244A^* were similar in the presence of untagged or YFP-tagged Z-α_1_-antitrypsin, exonerating the fluorescent protein tag from driving reduced calreticulin mobility (fig. S3C). Next, to assess whether the effect of calreticulin overexpression translated to other polymerogenic serpin family proteins, the mobility of mEmerald-tagged neuroserpin*^G392E^* was assessed by two-color ID-FRAP. When coexpressed with mCherry-KDEL, mEmerald-tagged neuroserpin*^G392E^* showed a spread of mobilities (11% immobile, 54% semi-mobile, and 35% mobile), similar to that seen for YFP-Z (Fig. 4H versus Fig. 4C), while coexpressed mCherry-KDEL was recovered within 1 s after bleach (Fig. 4I). Upon overexpression of calreticulin, neuroserpin*^G392E^* adopted the immobile phenotype in all cells analyzed (Fig. 4H) and was accompanied by slow recovery of HaloTag-calreticulin fluorescence (Fig. 4J), indicating a generalizable effect of calreticulin on immobilization of polymerogenic serpins in the ER.

Together, these data show that calreticulin mobility is retarded through confinement imposed by an immobile matrix of Z-α_1_-antitrypsin. As calreticulin chaperone activity is implicated in the immobilization of Z-α_1_-antitrypsin, it is likely that the Z-α_1_-antitrypsin immobilization observed during ER stress (Fig. 4, A and B) is, at least in part, the result of calreticulin up-regulation by ATF6 (fig. S4). The more pronounced effect on mobility of calreticulin and large protein assemblies (Fig. 2, F to N) compared with a small ER marker protein (Fig. 2, A to E, and figs. S2 and S3, A and B) indicates a size-dependent filtration effect imposed by the immobile Z-α_1_-antitrypsin matrix.

### Z-α_1_-antitrypsin immobilization is associated with larger polymeric species

**Fig. 5.**
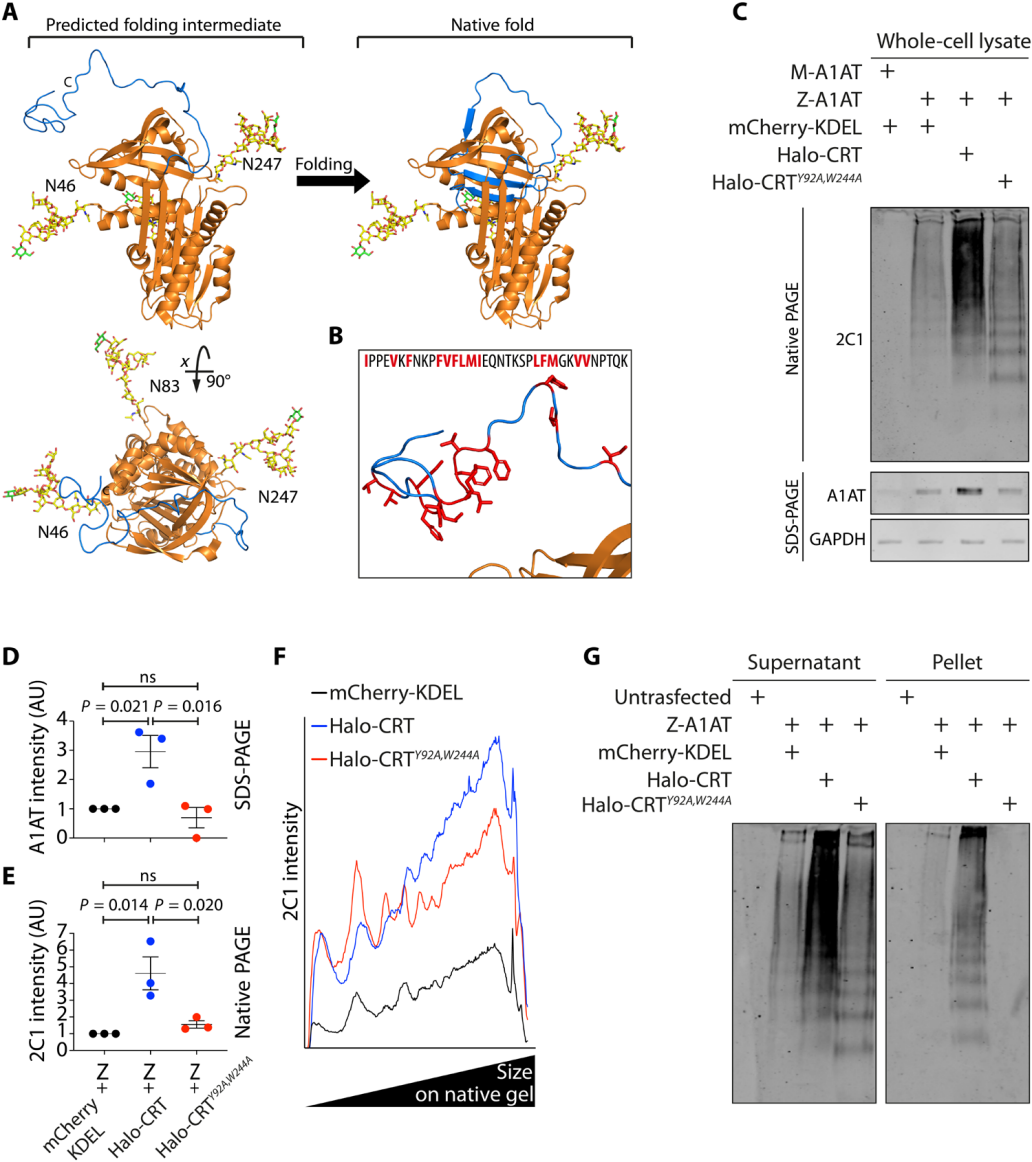
Calreticulin promotes larger Z-α_1_-antitrypsin polymer-containing species. (**A**) A structural model of a predicted late folding intermediate of Z-α_1_-antitrypsin, showing three Glcα1–3Manα1–2Manα1–2Man glycans at residues N46, N83, and N247. The C-terminal region is shown in blue. (**B**) An enlargement of the C terminus shows hydrophobic amino acid side chains in red (and in inset peptide sequence). (**C**) Whole-cell lysates of CHO-K1 cells transiently transfected to express untagged M- or Z-α_1_-antitrypsin (M- or Z-A1AT) with mCherry-KDEL and Z-A1AT with either HaloTag-calreticulin (Halo-CRT) or HaloTag-calreticulin*^Y92A,W244A^* (Halo-CRT*^Y92A,W244A^*) were separated by native-PAGE, and Western blots were probed with the α_1_-antitrypsin polymer-specific mAb_2C1_. The same samples were separated by SDS-PAGE and blotted for total α_1_-antitrypsin and glyceraldehyde-3-phosphate dehydrogenase (GAPDH) as a loading control. Quantitation was performed on (**D**) total α_1_-antitrypsin on SDS-PAGE and (**E**) total lane intensity of native-PAGE mAb_2C1_ signal, from three independent experiments, with means and SEs shown. (**F**) mAb_2C1_ signal intensity quantification on the blot shown in (C) was profiled from bottom to top of the gel. The *X* axis reflects increasing polymer size. (**G**) CHO-K1 cell lysates prepared as in (C) were centrifuged at 16,100*g* before both supernatant and pellet fractions were separated by native-PAGE. Western blots were probed with polymer-specific mAb_2C1_ antiserum. Representative gel of three experiments.

The notable effect of calreticulin expression on Z-α_1_-antitrypsin mobility led us to investigate mechanistic drivers of this process. Polymerization of Z-α_1_-antitrypsin is thought to occur via a C-terminal domain swap (*42*, *43*), proceeding via a late folding intermediate of the native folding pathway that is stabilized by the E342K Z-mutation (*44*). Accordingly, this folding intermediate is predicted to have an unfolded C-terminal domain (Fig. 5A, shown in blue), consisting of s1C, s4B, and s5B that are yet to insert into the pocket residing behind β sheet A (*45*). A structural model of this intermediate showed that the unfolded C-terminal domain would likely have the flexibility to come within close proximity to all three N-linked glycans of antitrypsin and hence could plausibly interact with calreticulin bound at these positions (Fig. 5A). Furthermore, the unfolded C terminus is populated with hydrophobic residues (Fig. 5B) that represent a plausible target for chaperone binding (*46*). We therefore hypothesized that increased calreticulin levels, leading to a higher frequency of chaperone-binding events, might stabilize a polymerogenic intermediate and thus promote polymerization. Z-α_1_-antitrypsin polymers can be visualized by native polyacrylamide gel electrophoresis (PAGE) Western blot using the conformation-specific antibody mAb_2C1_ (*47*). Polymer analysis by native PAGE was carried out on lysates of CHO-K1 cells expressing untagged Z-α_1_-antitrypsin with various other proteins (Fig. 5C). Cells coexpressing mCherry-KDEL with M-α_1_-antitrypsin produced negligible mAb_2C1_ reactivity compared to those expressing mCherry-KDEL with Z-α_1_-antitrypsin, where a ladder of mAb_2C1_-immunoreactive polymers was seen (Fig. 5C). Coexpression of calreticulin led to a significant increase in both total cellular Z-α_1_-antitrypsin (Fig. 5, C and D) and of Z-α_1_-antitrypsin polymer accumulation, which was greatly diminished in cells expressing the nonfunctional mutant calreticulin*^Y92A,W244A^* (Fig. 5, C and E). Furthermore, calreticulin-induced mAb_2C1_ reactive polymers dominated the upper region of the gel, indicating that they represent larger protein species (Fig. 5F). Centrifugation of lysates was then used to separate soluble and insoluble material, and only cells overexpressing calreticulin showed significant polymer accumulation in the pellet (Fig. 5G). Notably, lower–molecular weight polymers, which were similarly abundant in whole-cell lysates of cells expressing wild-type or mutant calreticulin (Fig. 5, C and F), were pelleted by centrifugation only from cells overexpressing wild-type calreticulin (Fig. 5G). This suggests that shorter-length polymers are also retained within the immobile Z-α_1_-antitrypsin matrix even upon cell lysis.

These data indicate that increased calreticulin levels, which promote Z-α_1_-antitrypsin immobilization, give rise to larger polymer-containing species that retain ER proteins. Next, we chose to investigate the physical properties of immobile Z-α_1_-antitrypsin.

### Z-α_1_-antitrypsin undergoes transition to a solid state in the ER lumen

**Fig. 6.**
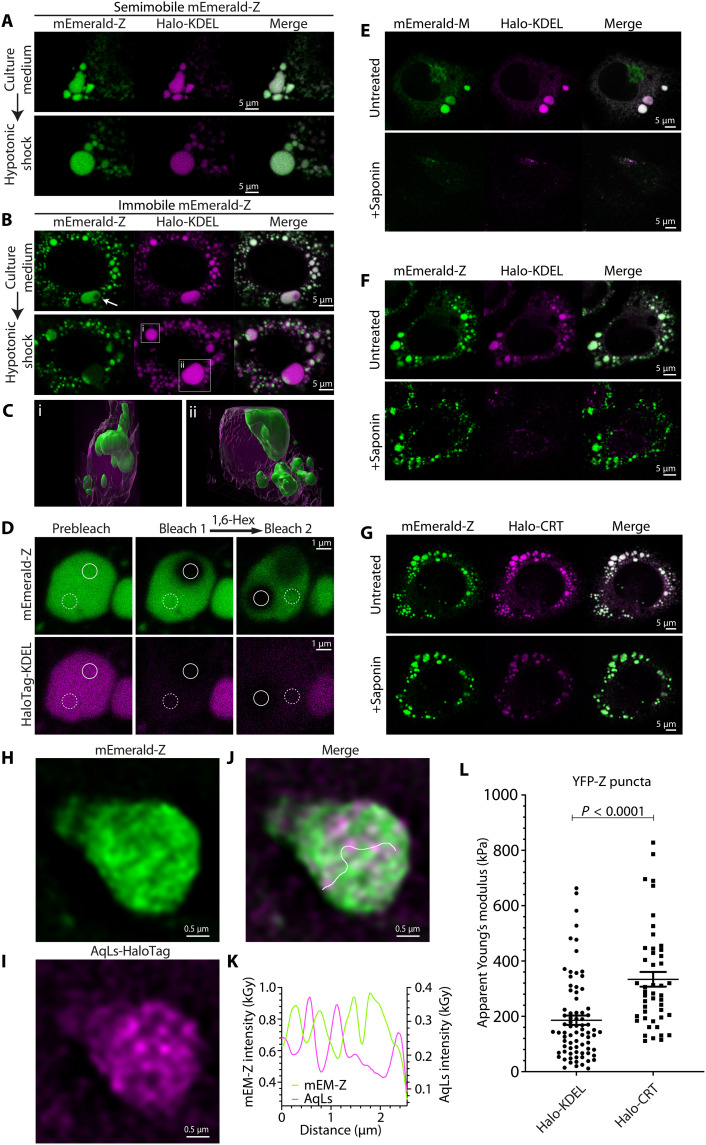
Immobile Z-α_1_-antitrypsin forms a solid matrix. CHO-K1 cells expressing mEmerald-tagged Z-α_1_-antitrypsin (mEmerald-Z) and HaloTag-KDEL (Halo-KDEL) labeled with TMR HaloTag ligand were analyzed by ID-FRAP to assign α_1_-antitrypsin mobility phenotype. (**A**) Semi-mobile mEmerald-Z or (**B**) immobile mEmerald-Z inclusions were imaged before (top) and after (bottom) 5 min of treatment with hypotonic buffer, leading to ER swelling. Images are representative of three independent experiments. Example inclusions marked “i” and “ii” are expanded in (**C**), as three-dimensional renderings from *Z*-stacked confocal image series, 7 min after hypotonic shock. (**D**) CHO-K1 cells were transiently transfected with expression plasmids encoding mEmerald-Z and HaloTag-KDEL and were analyzed by ID-FRAP. Images show ER inclusions of immobile mEmerald-Z before (left) and immediately after photobleach (middle). Cells were subsequently treated with 4% (w/v) 1,6-hexanediol (1,6-Hex) for 20 min before ID-FRAP assessment of Z-α_1_-antitrypsin mobility in the same inclusion (right). Images are representative of all 14 cells analyzed over three experiments. (**E** to **G**) CHO cells expressing (E) mEmerald-M and Halo-KDEL, (F) mEmerald-Z and Halo-KDEL, and (G) mEmerald-Z and Halo-CRT were imaged before (top) and after (bottom) saponin treatment. (**H** to **J**) Lattice SIM images of mEmerald-Z puncta detergent-extracted from cells coexpressing AqLs-HaloTag labeled with JF646 ligand. Images were reconstructed using the Zeiss SIM^2^ algorithm. The white line overlaid on the merged channel image (J) represents a linear ROI used to produce the histogram of fluorescence intensity gray values [kilogray (kGy)] along the length of the ROI (distance) shown in (**K**). (**L**) Apparent Young’s moduli of YFP-Z puncta extracted from cells expressing YFP-Z with either Halo-KDEL or Halo-CRT, assessed by atomic force microscopy (AFM) on a glass substrate. *P* value was assigned by Student’s *t* test.

Formation of immobile Z-α_1_-antitrypsin could plausibly be attributed to several phenomena. These include agglomeration of Z-α_1_-antitrypsin polymers with unfolded/misfolded proteins, changes to polymer organization or structure, or an increase in macromolecular crowding in the ER resulting from impaired protein export or degradation. To investigate these possibilities, we used hypotonic shock to induce osmotic swelling of the ER and thereby reduce macromolecular crowding (*48*). The mobility of mEmerald-tagged Z-α_1_-antitrypsin was assessed by ID-FRAP (fig. S5A) before hypotonic shock. Improved photostability of mEmerald over YFP aided its continuous imaging during hypotonic-driven ER dilatation. Osmotic swelling of semi-mobile Z-α_1_-antitrypsin inclusions led to homogeneous dispersion of both mEmerald-Z and an ER-marker protein HaloTag-KDEL throughout the expanded ER volume (Fig. 6A). This was also seen in rare cells with inclusions of mEmerald-M (fig. S5B). However, in cells with immobile mEmerald-Z inclusions, while ER swelling was again accompanied by homogeneous dispersion of HaloTag-KDEL, mEmerald-Z remained in discrete structures (Fig. 6B and movie S2). During swelling, immobile mEmerald-Z puncta appeared to remain tethered to the inclusion membrane, suggesting possible interaction with constituents of the ER membrane (Fig. 6C), but the puncta did not disperse or dissolve. These observations indicated that the immobility of Z-α_1_-antitrypsin in inclusions is not caused by tight packing of accumulated Z-α_1_-antitrypsin, confined by the ER membrane, but rather that Z-α_1_-antitrypsin undergoes a physical state change from a monodispersed to a solid condensed phase.

Material states that result in low protein mobility within cells include liquid-liquid phase separations (LLPSs) and transitions to solid states such as hydrogels, soft glasses, or crystalline aggregates (*49*). LLPS was deemed unlikely to account for Z-α_1_-antitrypsin immobilization, as immobile mEmerald-Z was unaffected by treatment with the small aliphatic alcohols 1,6-hexanediol (Fig. 6D) or propylene glycol (fig. S6A), which disrupt many LLPS protein condensates in cells (*50*). By contrast, green fluorescent protein (GFP)–tagged fused in sarcoma (FUS-GFP) LLPS condensates formed in the nucleus upon mild hypertonic stress readily dissolved under these conditions (fig. S6, B and C) (*51*, *52*). Next, cells expressing mEmerald-tagged α_1_-antitrypsin were treated with the detergent-like compound saponin, which partially permeabilizes cellular membranes. Saponin treatment led to a rapid ER depletion of both mEmerald-M and the small ER lumenal marker HaloTag-KDEL, as expected for soluble proteins (Fig. 6E). By contrast, mEmerald-Z remained within structures that morphologically resembled their parent ER inclusions despite ER membrane solubilization (as indicated by loss of HaloTag-KDEL fluorescence) (Fig. 6F). In cells expressing mEmerald-Z and HaloTag-calreticulin, both proteins endured upon saponin treatment (Fig. 6G), consistent with retention of calreticulin within the Z-α_1_-antitrypsin matrix (Fig. 4D). These observations are consistent with formation of a continuous solid phase of Z-α_1_-antitrypsin that is sufficiently porous to allow the transit of small proteins but traps HaloTag-calreticulin.

Confocal images of saponin-treated mEmerald-Z inclusions suggested underlying structure within the retained solid matrix [Fig. 6, F and G (bottom)]. To assess how matrix organization might influence protein complex distribution within inclusions, mEmerald-Z was coexpressed in CHO cells with ER-localized AqLs fused to HaloTag, labeled with the bright, photostable, and fluorogenic ligand JF646. Lattice SIM superresolution microscopy was performed on saponin-treated cells, and images of inclusions were reconstructed using Zeiss’ SIM^2^ algorithm (Fig. 6, H to J). Strong anticorrelation was observed between mEmerald-Z and AqLs-HaloTag fluorescence intensity, implying that large protein complexes (with dimensions of approximately 20 nm) may become trapped between high-density regions of the Z-α_1_-antitrypsin solid matrix (Fig. 6K).

As calreticulin overexpression increased the unit size of Z-α_1_-antitrypsin species observed by native PAGE (Fig. 5, C to G), we hypothesized that this could plausibly affect the mechanical properties of the Z-α_1_-antitrypsin solid matrix. To explore this, puncta of solid Z-α_1_-antitrypsin were liberated from cells by detergent, purified by differential centrifugation, before assessing their mechanical properties using atomic force microscopy (AFM). Force curves of isolated YFP-Z puncta, deposited on a glass substrate, were obtained (fig. S7). YFP-Z puncta extracted from cells coexpressing HaloTag-calreticulin showed increased stiffness compared to those extracted from cells coexpressing HaloTag-KDEL (Fig. 6L). These data indicate that calreticulin not only promotes formation of the solid phase of Z-α_1_-antitrypsin but also modulates the mechanical properties of the solid matrix to a more rigid conformation.

### Z-α_1_-antitrypsin mobility is reduced in ER tubules in a stress-induced manner

**Fig. 7.**
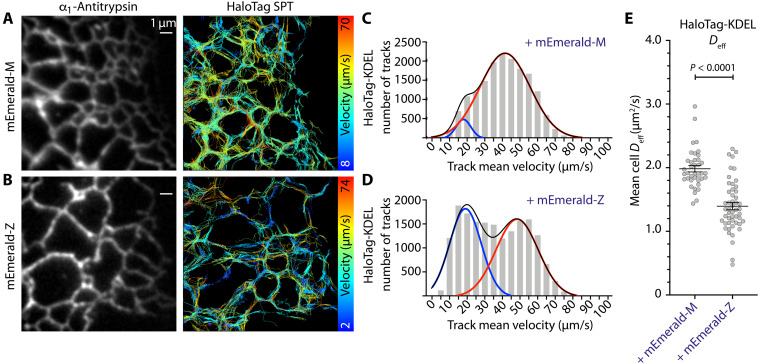
Single-particle tracking reveals reduced mobility in the tubular ER of Z-α_1_-antitrypsin–expressing cells. COS7 cells were transfected with mEmerald-tagged α_1_-antitrypsin and HaloTag-KDEL labeled with PA-JF646 ligand. Shown are fluorescence intensity micrograph of [(**A**), left] mEmerald-M or [(**B**), left] mEmerald-Z alongside projections of single-particle tracks (SPTs) acquired over 15,000 frames (corresponding right panels). Tracks are color-coded by mean track velocity. Frequency distribution histograms show mean track velocity of HaloTag-KDEL molecules collated from (**C**) 38 cells expressing mEmerald-M and (**D**) 50 cells expressing mEmerald-Z, fitted with a bimodal Gaussian distribution model color-coded for the low velocity peak (blue), high velocity peak (red), and combined fit (black). (**E**) Mean effective diffusion coefficients (*D*_eff_) of HaloTag-KDEL particles in each cell analyzed in (C) and (D), with mean and SE shown. *P* values were assigned by Student’s *t* test.

While ER inclusions are a hallmark of liver biopsies from Pi*ZZ individuals, many hepatocytes remain morphologically normal with no inclusions (*6*), generally assumed to represent a healthy pool of cells. However, the influence of Z-α_1_-antitrypsin expression on the reticular ER network has not been formally addressed. To investigate this, we optimized a single-particle tracking methodology for assessing protein mobility in tubular ER networks (*48*). Experiments were performed in COS7 cells, which are more resistant to ER fragmentation than CHO-K1 cells despite accumulation of mAb_2C1_-immunoreactive Z-α_1_-antitrypsin polymers (*47*). Cells were made to express either mEmerald-M or mEmerald-Z, and the mobility of the small inert protein HaloTag-KDEL was assessed using the photoactivatable far-red fluorescent HaloTag ligand PA-JF646 (*53*). Images were acquired at a frame rate of 167 Hz with sufficiently low HaloTag-KDEL particle density to operate in a single-molecule regime. Fidelity of spot detection and track assignment were assessed by simultaneously imaging the ensemble fluorescence of ER-localized mEmerald (fused to α_1_-antitrypsin or KDEL) to scaffold ER structure, revealing that HaloTag-KDEL tracks consistently mapped to the ER and ER tubules remained within the focal plane of imaging (Fig. 7, A and B, and movie S3). Mean track velocities of HaloTag-KDEL molecules were assessed in sections of predominantly tubular ER in cells expressing mEmerald-M or mEmerald-Z. The distributions of track velocities appeared bimodal, with peaks at approximately 20 and 45 μm/s (Fig. 7, C and D). Expression of mEmerald-Z produced a pronounced increase in the proportion of low-velocity tracks compared to cells expressing mEmerald-M [Fig. 7, A and B (right), and C and D], equating to a reduction in mean cell *D*_eff_ of HaloTag-KDEL from 2.0 μm^2^/s in cells expressing mEmerald-M to 1.4 μm^2^/s in cells expressing mEmerald-Z (Fig. 7E). These data indicate that Z-α_1_-antitrypsin restricts movement of ER proteins in tubular ER and in ER inclusions.

**Fig. 8.**
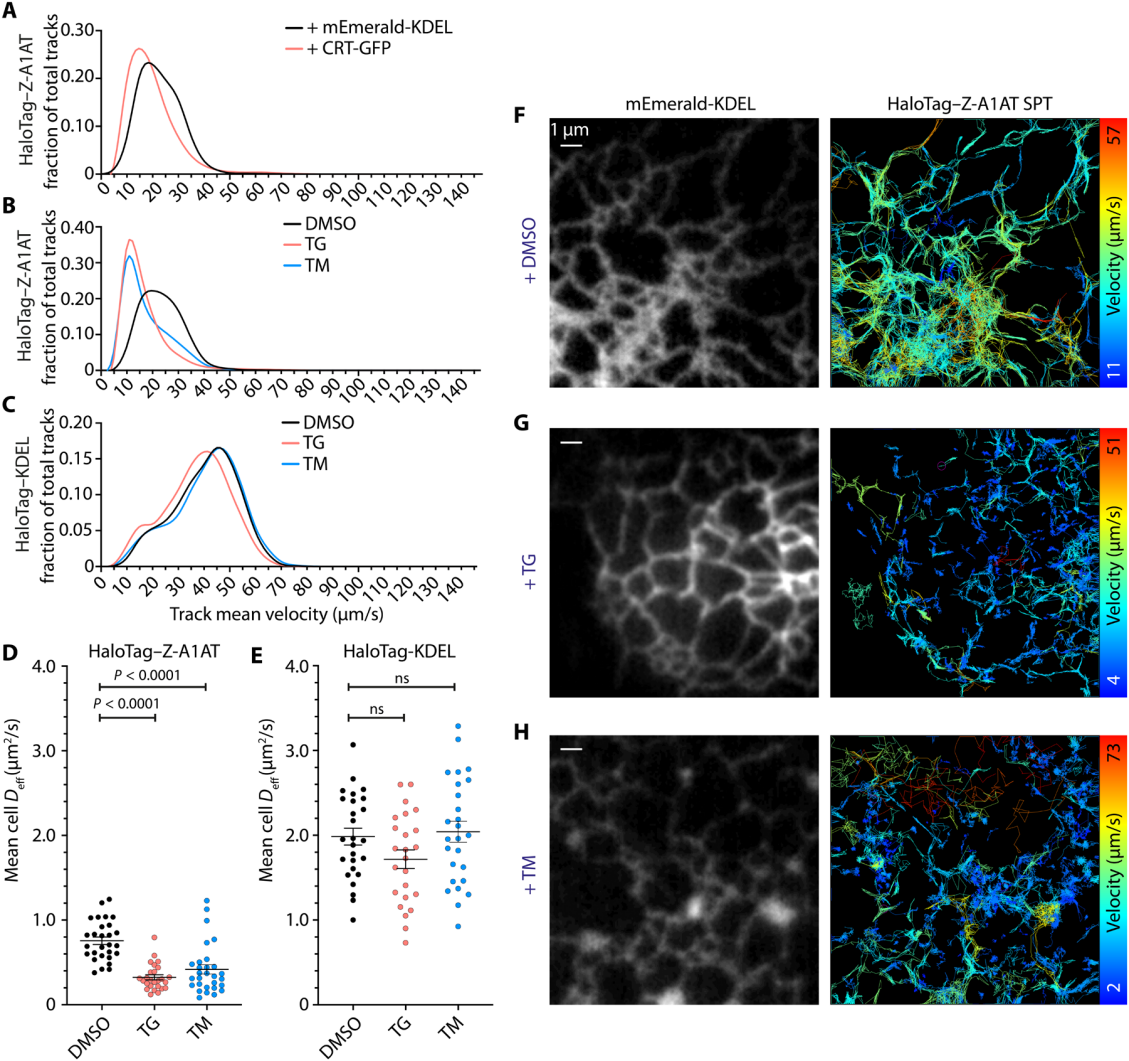
ER stress–induced Z-α_1_-antitrypsin immobilization occurs in the tubular ER network. Single-particle tracking of PA-JF646–labeled HaloTag–Z-α_1_-antitrypsin (HaloTag–Z-A1AT) was performed in COS7 cells. (**A**) Spline fit of mean track velocity histograms of HaloTag–Z-A1AT in cells coexpressing mEmerald-KDEL or calreticulin tagged with GFP (CRT-GFP). (**B**) Spline fit of mean track velocity histograms of HaloTag–Z-A1AT coexpressed with mEmerald-KDEL, treated for 8 hours with either DMSO, 0.02 μM TG, or TM (2 μg/ml). PA-JF646 labeling of HaloTag–Z-A1AT was performed before addition of ER stress inducers, ensuring that labeled HaloTag–Z-A1AT glycosylation state would be unaffected by tunicamycin. (**C**) Spline fit of mean track velocity histograms of PA-JF646–labeled HaloTag-KDEL molecules in cells treated for 8 hours with either DMSO, 0.02 μM TG, or TM (2 μg/ml). HaloTag-KDEL was labeled immediately before imaging. Effective diffusion coefficients (*D*_eff_) for (**D**) HaloTag–Z-A1AT and (**E**) HaloTag-KDEL were extracted from the data presented in (B) and (C), respectively. Images showing representative cells were analyzed in (B) and (D), treated with (**F**) DMSO, (**G**) TG, or (**H**) TM. Left-hand images show mEmerald-KDEL ER marker, and right-hand images show tracks of PA-JF646–labeled HaloTag–Z-A1AT molecules color-coded by mean velocity. Minimum number of cells analyzed for each treatment was (A) 15, (B) 28, and (C) 24, over a minimum of three independent experiments. *P* values were assigned by Student’s *t* test.

We next assessed the effect of chaperone overexpression on the mobility of Z-α_1_-antitrypsin itself in the tubular network, by analyzing motion of HaloTag–Z-α_1_-antitrypsin particles. Recapitulating the effects seen in ER inclusions, the mean track velocity of HaloTag–Z-α_1_-antitrypsin particles was reduced both during overexpression of calreticulin (Fig. 8A) and after 8 hours of ER stress induction (Fig. 8B and movies S4 and S5). By contrast, ER stress induction alone did not affect the mobility of the inert protein HaloTag-KDEL (Fig. 8C). These relationships were confirmed by assessment of protein effective diffusion coefficients extracted from the mean instantaneous velocity of tracked particles in each cell analyzed (Fig. 8, D and E). Similar architecture of tubular ER in stressed and unstressed cells suggested that the observed effects on protein mobility are unlikely to be dominated by altered network geometries but rather by changes to ER lumenal biophysics (Fig. 8, F to H; note the shortened, slower tracks of Halo-Z-A1AT in stressed ER). These data parallel our findings using ID-FRAP and FCS in ER inclusions. Furthermore, the notion that Z-α_1_-antitrypsin also reduces mobility of ER proteins in the tubular ER network suggests that hepatocytes of Pi*ZZ individuals with morphologically normal ER may represent a population relevant to liver pathology. Moreover, calreticulin overexpression, as well as stress induction that drives up-regulation of chaperones including calreticulin, reduces movement of Z-α_1_-antitrypsin itself. Together, these data indicate that Z-α_1_-antitrypsin immobilization is likely to initiate in the morphologically normal tubular ER, which represents a significant proportion of the ER in the hepatocytes of α_1_-antitrypsin–deficient individuals.

## DISCUSSION

Here, we show that Z-α_1_-antitrypsin undergoes a phase transition to a solid state within the lumen of the ER, leading to molecular filtration of ER proteins in a size-dependent manner. While many examples of phase-state transitions have been shown in living cells, interpretation of their biological consequences is achieved less frequently (*51*, *52*, *54*, *55*). The filtration of soluble proteins within the lumenal space of a cellular organelle represents a previously undescribed mechanism through which a change in protein phase state can influence cellular processes. This observation has potential significance to a wide range of pathologies. Relevant to the ER, FENIB arises from mutations in neuroserpin that lead to an autosomal dominant form of dementia, characterized by the accumulation of protein polymers in a fragmented ER network in neurons (*56*, *57*). Here, we show that molecular filtration in the ER is likely a consequence of the disease-associated mutant neuroserpin*^G392E^* (Fig. 4, H to J). Furthermore, in diabetes insipidus, specific mutants of vasopressin form fibrillar aggregates that accumulate within the ER, distorting its morphology (*58*). The extent to which molecular filtration is involved in this and other disease states is a question for future studies.

The folding pathway of α_1_-antitrypsin is altered by the E342K Z-mutation, which is thought to prolong occupancy of a folding intermediate conformation through which polymerization is favored (*43*, *59*). This likely involves delayed insertion of the C-terminal domain into a pocket at the B-C barrel, arising from local destabilizing effects of E342K (Fig. 5A) (*45*). Of the mechanisms proposed for Z-α_1_-antitrypsin polymerization, compelling evidence supports a domain swap wherein the C-terminal domain of one protomer inserts into its native fold site of a second protomer (*42*, *43*, *45*), likely promoted by the exceptionally high endogenous expression levels of α_1_-antitrypsin in hepatocytes (*60*). The C-terminal domain contains a high proportion of hydrophobic residues (Fig. 5B) that facilitate its engagement in the native (or domain swapped) structure (*43*). Hence, an exposed C-terminal domain conformation could plausibly be stabilized by ER chaperone interactions, which occur at exposed hydrophobic peptide sequences (*61*–*63*). It is probable that increased chaperone levels would prolong the duration of an unfolded and exposed C-terminal domain, as chaperone binding is thought to stabilize non-native conformations (*64*). Structural modeling of α_1_-antitrypsin reveals intriguing features relating to the positioning of putative glycosylation sites relevant to lectin chaperone interactions (*65*–*67*). In addition, the glycosylation site at N46 is positioned at the opening of the hydrophobic cavity that accommodates β sheet B in the final stages of folding (Fig. 5A). Calreticulin binding at this site could plausibly delay β sheet B completion either by direct binding of the hydrophobic C-terminal peptide sequence or by affecting the structure of the B-C barrel that accepts the C terminus in the native fold. Calreticulin binds to hydrophobic peptide sequences providing protection from aggregation (*46*, *68*, *69*), reportedly mediated through binding sites both proximal (*41*) and distal (*70*) to glycan binding, in addition to recruiting the chaperones ERp57 (*71*) and ERp27 (*72*). Furthermore, sequence analysis of the C-terminal 20 residues of α_1_-antitrypsin reveals a number of predicted binding sites for the ER-localized Hsp70 family chaperone, BiP (*73*), suggesting that calreticulin interaction might cooperate with other ER chaperones in prolonging residency of the polymerogenic folding intermediate state. Notably, calreticulin has an ER membrane–anchored homolog, calnexin, which also interacts with substrates via N-linked glycans. While membrane localization of calnexin would prevent it from accessing regions of the Z-α_1_-antitrypsin solid matrix distal to the ER membrane, it is plausible that calnexin may also promote polymerization proximal to the ER membrane.

The UPR maintains ER proteostasis by balancing the load of unfolded client proteins with the chaperone machinery required to fold them (*10*). The expression of Z-α_1_-antitrypsin is not accompanied by strong induction of the UPR (*11*–*13*), supporting the notion that polymers of Z-α_1_-antitrypsin are assemblies of well-folded proteins that do not sequester a substantial pool of chaperones. However, the immobile matrix of Z-α_1_-antitrypsin, promoted by calreticulin, retarded the movement of ER proteins including calreticulin itself. Given that calreticulin can interact with substrates in both a glycan-dependent and glycan-independent manner (*74*), we cannot exclude the possibility that direct binding of the chaperone to immobile Z-α_1_-antitrypsin contributes to reduced HaloTag-CRT*^Y92A,W244A^* mobility in the presence of immobile YFP-Z. However, our data describing reduced mobility of inert proteins (HaloTag-KDEL and ER-AqLs) better supports a model whereby calreticulin mobility is reduced via molecular filtration by a solid matrix formed of Z-α_1_-antitrypsin polymers. We propose that this effect is likely to be further enhanced by the increased stiffness of the Z-α_1_-antitrypsin solid matrix that is promoted by calreticulin (Fig. 6L). Protein folding is governed by diffusion at both the intramolecular level, as nascent chains fold to their native conformation, and the intermolecular level, as chaperones and client proteins interact through random collisions. Hence, chaperone immobilization could plausibly alter protein folding efficiency in the ER leading to ER stress. Our data suggest that in cells expressing polymerogenic antitrypsin, up-regulation of chaperones during ER stress may further compromise ER protein folding in a positive feedback mechanism by immobilizing ER chaperones within the solid Z-α_1_-antitrypsin matrix (fig. S8). This model provides a rationale for the heighted sensitivity of Z-α_1_-antitrypsin–expressing cells to ER stress despite their absence of basal UPR activation (*12*, *13*).

The observations reported here do not exclude a protective role for the encapsulation of Z-α_1_-antitrypsin within ER inclusions, as occurs for the heavy chain of immunoglobulin M (μ_s_), which, in the absence of its light-chain binding partner, accumulates in dilated ER, reminiscent of Z-α_1_-antitrypsin inclusions (*75*). This accumulation of μ_s_ appears to be well tolerated, so immobilization of Z-α_1_-antitrypsin, promoted by the UPR, might potentially corrupt a protective mechanism of the proteostatic machinery. Our observation that ER stress reduces Z-α_1_-antitrypsin mobility in ER tubules (Fig. 8, B and D) is compatible with a model of protective ER vesiculation, which would be predicted to reduce confinement of proteins in the ER. The survival of cells that show a high degree of ER vesiculation indicates that ER function is maintained despite these profound structural changes.

Our findings offer an explanation for the hypersensitivity to ER stress experienced by Z-α_1_-antitrypsin–expressing cells, suggesting that a positive feedback relationship exists between ER stress and the solidification of Z-α_1_-antitrypsin polymers. Accordingly, physiological ER stress in hepatocytes provokes a UPR that initially promotes up-regulation of chaperones, including calreticulin, that serve to ameliorate the stress. However, in this context, an increased abundance of calreticulin promotes solidification of Z-α_1_-antitrypsin polymers via a mechanism that requires Z-α_1_-antitrypsin glycosylation. The result of polymer solidification is the immobilization of ER proteins, including the ER chaperones required to resolve ER stress.

Our discovery that a transcriptional output of the UPR influences the physical state of accumulating Z-α_1_-antitrypsin poses an opportunity to design novel therapeutic strategies with which to combat α_1_-antitrypsin deficiency. Understanding the relevance of this disease mechanism to other proteinopathies, both in the ER and other compartments, can provide insight into a broad spectrum of disease states.

## MATERIALS AND METHODS

### Plasmids, antibodies, and fluorescent labels

Mammalian expression plasmids encoding α_1_-antitrypsin tagged at its N terminus with either YFP or HaloTag, separated by a flexible linker, were generated from pcDNA3.1 constructs encoding α_1_-antitrypsin, as described previously in (*16*). α_1_-antitrypsin N-terminally tagged with mEmerald was generated by replacing the YFP coding sequence of YFP-M and YFP-Z with that of mEmerald by Gibson assembly. The glycosylation-null variant of α_1_-antitrypsin was made by Gibson assembly to insert a synthesized gene fragment (GeneArt, Thermo Fisher Scientific, USA) encoding the region between H67 and A274 of unprocessed α_1_-antitrypsin, containing N70A, N107A, and N271A substitutions (N46A, N83A, and N247A in the signal peptide-cleavage protein and named as such here). ER-AqLs-Sapphire was made by synthesizing a gene fragment (Twist Bioscience, USA) encoding the signal sequence of human preprolactin upstream of the AqLs-coding sequence, codon-optimized for mammalian expression. This fragment was used to replace the PfV coding sequence in pCMV-PfV-Sapphire-IRES-DsRed [pLH1337 (*31*); Liam Holt laboratory, New York University]. ER-AqLs–HaloTag was generated by replacing Sapphire with HaloTag by Gibson assembly. Plasmids encoding mCherry-KDEL (*16*), HaloTag-KDEL (*21*), FUS-GFP (*76*), HaloTag fused to human calreticulin (*48*), and mEmerald-KDEL (*77*) were reported previously. HaloTag-calreticulin*^Y92A,W244A^* was generated by site-directed mutagenesis of HaloTag fused to human calreticulin. Calreticulin-GFP was expressed from a plasmid encoding rat calreticulin C-terminally fused to GFP (*78*). A list of primers and plasmids can be found in table S1.

Antibodies used in this study were raised against total α_1_-antitrypsin (A0409, Sigma-Aldrich), glyceraldehyde-3-phosphate dehydrogenase (2118, Cell Signaling Technology), and the α_1_-antitrypsin polymer–specific mAb_2C1_ (HM2289, Hycult Biotech). HaloTag ligands JF646 (*53*) and PA-JF646 (*79*) were a gift from the Luke Lavis laboratory (Janelia, USA). TMR HaloTag ligand was purchased (Promega, USA).

### Mammalian cell culture

CHO-K1 cells were cultured as described previously (*16*), in F12 Ham nutrient mixture (Merck, Germany) supplemented with 10% fetal bovine serum (FBS) and GlutaMAX (Thermo Fisher Scientific, USA). Tet-On CHO-K1 lines with inducible expression of α_1_-antitrypsin (*16*) were cultured as for CHO-K1 cells, but supplemented with tetracycline-free FBS (PAN-Biotech, UK). COS7 cells (catalog no. 87021302-1VL, Sigma-Aldrich, UK) were cultured in Dulbecco’s modified Eagle’s medium with glucose (4500 mg/liter; Sigma-Aldrich, UK) supplemented with 10% FBS. Transient transfections in CHO-K1 cells were performed using Lipofectamine LTX (Thermo Fisher Scientific, USA) at a ratio of 4 μl per 1 μg of DNA and in COS7 cells using FuGENE 6 (Promega, USA) at a ratio of 3 μl per 1 μg of DNA.

### Measurement of ER microviscosity by ROVI

ROVI was performed as described previously (*21*). Briefly, CHO-K1 cells were seeded in eight-well glass bottom chamber slides (Lab-Tek II Chamber Coverglass) and transiently transfected 48 hours before imaging. Cells were labeled with 0.1 μM BODIPY-O_2_-HaloLigand (a molecular rotor based on meso-substituted boron dipyrrin) for 30 min in phosphate-buffered saline (PBS) before image acquisition by time-correlated single-photon counting. FLIM was performed using a confocal laser scanning microscope (Leica, SP5 II) with the Ti:sapphire laser in two-photon excitation mode operated at 880 nm. A PMC-100-1 photomultiplier tube (Hamamatsu) and an SPC-830 single-photon counting card (Becker & Hickl) were used for data acquisition. Fluorescence was collected between 500 and 580 nm. The instrument response factor was obtained by measuring second-harmonic generation signal from urea crystals on a glass cover slide.

### Intensity differential fluorescence recovery after photobleaching

A modified FRAP protocol, here named ID-FRAP, was performed on “large” ER inclusions, being those with a diameter greater than 1 μm. CHO-K1 cells were seeded at 5 × 10^4^ cells per 35-mm dish on 25-mm glass cover slips, and transfection was performed 2 to 6 hours later. Cells were imaged 48 hours after transfection. Fluorophores were excited at 488 nm (mEmerald), 514 nm (YFP), 561 nm [HaloTag-tetramethylrhodamine (TMR) and mCherry], and 633 nm (HaloTag-JF646) and were imaged using a Zeiss LSM 780 confocal microscope with GaAsP detectors using a ×63 1.4 numerical aperture (NA) oil immersion lens. Images were captured with 512 × 512 pixel frame size, line switching between channels, with a two-color frame acquisition time of 0.928 s. Photobleaching was achieved using excitation lasers at 100% power for 25 scan iterations. The circular bleached ROI was chosen to be approximately one-fourth of a diameter of the ER inclusion. An unbleached control ROI of the same dimensions was placed within the same inclusion approximately one ROI diameter away from the bleached ROI. An image series was acquired continuously and was ended between 80 and 120 s after bleach, often dictated by inclusion movement outside of the field of view. For quantitation purposes, inclusion movement during the period of imaging was corrected by adjustment of ROI position relative to the inclusion’s perimeter (fig. S9). After normalization to starting ROI intensities, the intensity differential (Δ*I*) between the two ROIs in a single inclusion, bleached versus unbleached control, was calculated (Δ*I* = Intensity^control^ − Intensity^bleached^) and was used to define the following categories of protein mobility: mobile, if the FRAP bleach did not cause an intensity difference larger than 10% of initial intensity (Fig. 3B); semi-mobile, if the bleach caused the intensity difference between the bleached and control region larger than 10% but, within 80 s after bleach, the intensity difference dropped below the homogenization threshold value of 10% (Fig. 3C); and immobile, if the intensity difference between the bleached and control region was greater than 10% and did not recover below this threshold within 80 s after bleach (Fig. 3D). Hence, ID-FRAP reported on intrainclusion protein mobility with minimal confounding influence from ER connectivity. ID-FRAP recovery times report the time taken for fluorescence intensity differential (Δ*I*) of a protein between control and bleach ROIs within the same inclusion to fall below 10%.

### Fluorescence correlation spectroscopy

All FCS measurements were performed with HaloTag proteins labeled with TMR ligand (Promega, USA). CHO-K1 cells were seeded at 5 × 10^4^ cells per 35-mm dish on 25-mm glass coverslips (high precision no. 1.5H, Marienfeld), and transfection were performed 2 to 6 hours later. Cells were imaged at the center of large ER inclusions 48 hours after transfection using a ×40 1.2 NA water objective on a Zeiss LSM 780 confocal microscope with Zen 2.6 software package (Black edition). Axial and lateral focal radii of the microscope point spread function (PSF) was calibrated using an aqueous solution of rhodamine B excited at 561 nm, calculated by Zen 2.6 software using a reference diffusion coefficient for rhodamine B of 602.6 μm^2^/s at 37°C (as reported in FCS application notes; PicoQuant, Germany). Before FCS measurement, cells were bleached by 514- and 561-nm lasers to reduce the signal to 10 to 200 kilocounts/s corresponding to a density of 3 to 350 particles within the PSF. FCS was performed using 0.1% laser power to minimize bleaching, producing 1 to 10 kilocounts/s per molecule. The parameters used for fitting of data were as follows: Lag time analyzed, 9.6 μs to 1.68 s; amplitude (fixed = 1); axial focus radius = 1.123 μm; lateral focus radius = 0.206 μm; and one component fit with anomalous mode of diffusion (anomalous parameter set free to be determined by the software). Zen 2.6 Black edition software generated the anomalous parameter and diffusion coefficient by fitting the experimental data to the function describing diffusion with set parameters (described above). As the anomalous parameter and diffusion coefficient were set “free,” the software manipulated these parameters and provided the values that gave the best fit of the data (*80*). While inclusions were selected to be large enough to fit the entire PSF volume within, the pronounced noise in residual traces (Fig. 2, B and C) is likely a product of measurement within a tightly confined regime enforced by the surrounding membrane of the ER inclusion. These operations were made according to the microscope usage manual issued by Zeiss, Germany.

The formula for fitting the autocorrelation curve was as follows

$$G(\tau )=1+A*G_{d}(\tau )$$

where *G_d_*(τ) is the translation and *A* is the amplitude.

Translation is calculated by

$$G_{d}(\tau )=\sum_{i=1}^{3}\frac{\Phi_{i}}{{\left(1+{\left(\frac{\tau }{\tau_{d,i}}\right)}^{\alpha_{i}}\right)}^{e_{d1}}*{\left({\left(1+{\left(\frac{\tau }{\tau_{d,i}}\right)}^{\alpha_{i}}*\frac{1}{S}\right)}^{0.5}\right)}^{e_{d2}}}$$

$$\Phi_{i}=\frac{f_{i}*{\eta_{i}}^{2}}{\sum_{i=1}^{3}{\left(f_{i}*\eta_{i}\right)}^{2}}$$

$$S=\frac{\omega_{z}}{\omega_{r}}$$

where *i* is the index of component (1,2,3), Φ*_i_* is the fractional intensity, *f_i_* is the fraction of molecules, η*_i_* is the molecular brightness, τ_*d*, *i*_ is the diffusional correlation time, *S* is the structural parameter, ω*_z_* is the axial focus radius (1.123 mm), ω*_r_* is the lateral focus radius (0.206 μm), and α*_i_* is the anomaly parameter.

For one-component diffusion (*i* = 1 and *f_i_* = 1) and the three-dimensional diffusion (*e*_*d*1_ = 1 and *e*_*d*2_ = 1)

$$\Phi =\frac{f*\eta^{2}}{{\left(f*\eta \right)}^{2}}=\frac{1*\eta^{2}}{{\left(1*\eta \right)}^{2}}=\frac{\eta^{2}}{\eta^{2}}=1$$

$$G_{d}(\tau )=\frac{1}{\left(1+{\left(\frac{\tau }{\tau_{d}}\right)}^{\alpha })*({\left(1+{\left(\frac{\tau }{\tau_{d}}\right)}^{\alpha }*\frac{1}{S}\right)}^{0.5}\right)}$$

Amplitude is calculated by

$$A=\frac{\gamma }{N}$$

where γ is the geometric factor for PSF (γ = 1 for cylindrical PSF) and *N* is the average number of molecules in the observation volume.

### Total internal reflection fluorescence microscopy imaging of AqLs

Total internal reflection fluorescence (TIRF) microscopy images were acquired using a Zeiss Elyra7 wide-field microscope using a ×63 1.46 NA oil immersion TIRF objective. Dual-channel synchronous capture was achieved using an OptoSplit beam splitter (Cairn Research Ltd., UK) and two pco.edge scientific complementary metal-oxide semiconductor (sCMOS) cameras (PCO, Germany). Fluorophores were excited at 488 nm (Sapphire, mEmerald) and 642 nm (HaloTag PA-JF646). While sapphire is only weakly excited at 488 nm (~5% of its excitation maximum), its assembly on the surface of AqLs GEMs significantly alters its photochemistry, facilitating specific excitation of assembled GEMs at this wavelength (*31*). AqLs GEMs were imaged with cameras free running at a frame rate of 50 Hz. Kymographs were generated using Fiji version 1.0 to analyze intensity profiles through linear ROIs.

### Single-particle tracking

COS7 cells were seeded at a density of 3 × 10^4^ cells per 35-mm dish on 25-mm 1.5H precision cover slips (Marienfeld, Germany), and transfection was performed 2 to 6 hours later. Cells were imaged 18 hours after transfection. TIRF microscopy images were acquired using a Zeiss Elyra7 wide-field microscope using a ×63 1.46 NA oil immersion TIRF objective. Dual-channel synchronous capture was achieved using an OptoSplit beam splitter (Cairn Research Ltd., UK) and two pco.edge sCMOS cameras (PCO, Germany). Fluorophores were excited at 488 nm (Sapphire, mEmerald) and 642 nm (HaloTag PA-JF646). Images were captured with an exposure time of 4 ms at a frame rate of 167 Hz. Photoactivation of PA-JF646 bound to HaloTag-KDEL was achieve by continuous exposure to 405-nm laser light to tune an appropriate density of particles within the 128 × 128 pixel frame to permit tracking (see movie S5 for particle density). A Laplacian of Gaussian filter was applied to HaloTagged protein images, and single spot detection was performed with subpixel localization. Particle tracks were assigned using the simple Linear Assignment Problem (LAP) tracker of the TrackMate ImageJ plugin (*81*), and a threshold minimum number of spots within a track was set to 30 to increase spot linking confidence. Mean track velocity was extracted for each assigned particle track and collated with equal weighting across all cells in an experimental group. The mean particle step size was calculated across all tracked particles in individual cells, from which the mean squared displacement was calculated. Mean effective diffusion coefficients were calculated from the mean step size of particles per cell, accounting for the mean localization precision error of imaging.

### Native and SDS-PAGE

CHO-K1 cells were grown to approximately 80 to 100% confluency in a six-well cell culture plate (Greiner Bio-One, UK). Wells were washed with 5 ml of PBS prechilled on ice. Two hundred microliters of ice-cold lysis buffer [10 mM Hepes, 50 mM NaCl, 560 mM sucrose, 0.5% (v/v) Triton, 0.4 mM phenylmethylsulfonyl fluoride, and 1× protease inhibitor cocktail (Roche, Switzerland)] was applied to each well, and the homogenate was collected. Cell lysates were sonicated in the water bath sonicator for 30 min at 4°C. One hundred microliters of the sonicated total cell lysate was centrifuged at 16,100*g* for 10 min in a bench-top centrifuge at 4°C, and supernatant was separated from pelleted material. The pellet was resuspended in 100 μl of the homogenization buffer by sonication in the water bath sonicator for 30 min at 4°C. Samples were separated by SDS-PAGE under reducing conditions and by native PAGE as described previously (*13*).

### Osmotic manipulation of cultured cells during imaging

Cells were prepared and imaged as described for FRAP experiments. Hypotonic swelling was achieved by addition of 6 volumes of Milli-Q water to 1 volume of culture medium to achieve an osmolality of 44 mosmol/kg. Images were acquired 5 min after addition of hypotonic buffer.

### Lattice SIM

Lattice SIM imaging was performed using a Zeiss Elyra7 microscope using a ×63 1.4 NA oil immersion objective. mEmerald and HaloTag labeled with JF646 were excited at 488 and 642 nm, respectively, and images were captured simultaneously with 40-ms exposure time using an OptoSplit beam splitter and two pco.edge sCMOS cameras. Cells were imaged live, and *Z*-stacks were acquired with 55-nm sectioning. Images were processed using Zeiss’ SIM^2^ algorithm in three dimensions using the “standard – live” settings with the sectioning set at 92 and intensity scaled with the original image. A linear background subtraction was performed on both channels of the processed image, and pixel number was increased fourfold. Linear ROI intensity was measured using Fiji (ImageJ).

### AFM of purified YFP-Z puncta

YFP-Z puncta were extracted from ER inclusions by incubation of CHO cells in lysis buffer [150 mM NaCl, 10 mM tris (pH 7.5), 0.5 mM EDTA, and 0.5% Triton X-100] containing cOmplete protease inhibitors (Roche, CH) 48 hours after transient transfection with expression vectors encoding YFP-Z and either HaloTag-KDEL or HaloTag-calreticulin and subjected to centrifugation at 13,000*g* for 15 min to isolate cellular debris. Supernatant was discarded, and the pellet was resuspended in fractionation buffer [50 mM NaCl, 560 mM sucrose, 10 mM Hepes (pH 7.5), and 0.5% Triton X-100] and spun twice at 800*g* for 5 min to remove nuclei. YFP-Z puncta were pelleted by centrifugation at 13,000*g* for 10 min and resuspended in resuspension buffer [150 mM NaCl and 20 mM Hepes (pH 7.5)], incubated for 16 hours at 4°C, before the sedimented material was discarded and, the supernatant fraction was retained for AFM analysis.

The samples were deposited on glass coverslips and incubated at room temperature for 15 min. The samples were then washed three times with Hepes buffer and loaded on the AFM sample holder. AFM measurements were performed on a Bioscope Resolve AFM (Bruker), operated in PeakForce QNM mode, using precalibrated PeakForce QNM-Live Cell probes, with an average spring constant of 0.07 N/m. The force curves were then fitted to a Hertz model, using NanoScope analysis, to extract the apparent Young’s modulus of the puncta.

### BODIPY-HaloLigand synthesis

BODIPY-HaloLigand (figs. S10 to S12) was synthesized as detailed in the Supplementary Materials.

## Notes

View/request a protocol for this paper from *Bio-protocol*.
