# Supplementary material for: Z-α1-antitrypsin polymers impose molecular filtration in the endoplasmic reticulum after undergoing phase transition to a solid state
Source: Sci Adv. 2022 Apr 8;8(14):eabm2094. doi: 10.1126/sciadv.abm2094 (PMC8993113; doi:10.1126/sciadv.abm2094)
Supplement: Supplementary file 1 — Supplementary Text Figs. S1 to S12 Table S1 [file sciadv.abm2094_sm.pdf]

Science Advances
AAAS

Supplementary Materials for

Z-α_1-antitrypsin polymers impose molecular filtration in the endoplasmic reticulum after undergoing phase transition to a solid state

Joseph E. Chambers*, Nikita Zubkov, Markéta Kubánková, Jonathon Nixon-Abell, Ioanna Mela, Susana Abreu, Max Schwiening, Giulia Lavarda, Ismael López-Duarte, Jennifer A. Dickens, Tomás Torres, Clemens F. Kaminski, Liam J. Holt, Edward Avezov, James A. Huntington, Peter St George-Hyslop, Marina K. Kuimova, Stefan J. Marciniak*

*Corresponding author. Email: jec202@cam.ac.uk (J.E.C.); sjm20@cam.ac.uk (S.J.M.)

Published 8 April 2022, *Sci. Adv.* **8**, eabm2094 (2022)
DOI: 10.1126/sciadv.abm2094

The PDF file includes:

Supplementary Text
Figs. S1 to S12
Legends for movies S1 to S5
Table S1

Other Supplementary Material for this manuscript includes the following:

Movies S1 to S5

Supplementary Text

1. Synthesis and compound characterisation

1.1 General Materials and Methods

The manipulation of all air and/or water sensitive compounds was carried out using standard inert atmosphere techniques. All chemicals were used as received from commercial sources without further purification. Anhydrous solvents were used as received from commercial sources. Analytical thin layer chromatography (TLC) was carried out on Merck® aluminium backed silica gel 60 GF254 plates and visualisation when required was achieved using UV light or I_2. Flash column chromatography was performed on silica gel 60 GF254 using a positive pressure of nitrogen with the indicated solvent system. Where mixtures of solvents were used, ratios are reported by volume. Nuclear magnetic resonance spectra were recorded on 300 MHz spectrometers at ambient probe temperature. Chemical shifts for ^{1}H NMR spectra are recorded in parts per million from tetramethylsilane with the solvent resonance as the internal standard ($CDCl_3$: δ = 7.26 ppm). ^{13}C NMR spectra were recorded with complete proton decoupling. Chemical shifts are reported in parts per million from tetramethylsilane with the solvent resonance as the internal standard ($^{13}CDCl_3$: 77.36 ppm). ^{19}F NMR spectra were recorded with complete proton decoupling. Chemical shifts are reported in parts per million referenced to the standard hexafluorobenzene: −164.9 ppm. Mass spectra were carried out using ElectroSpray Ionisation (ESI), and only molecular ions are reported. (Figure S10-12)

1.2 Synthetic Procedures

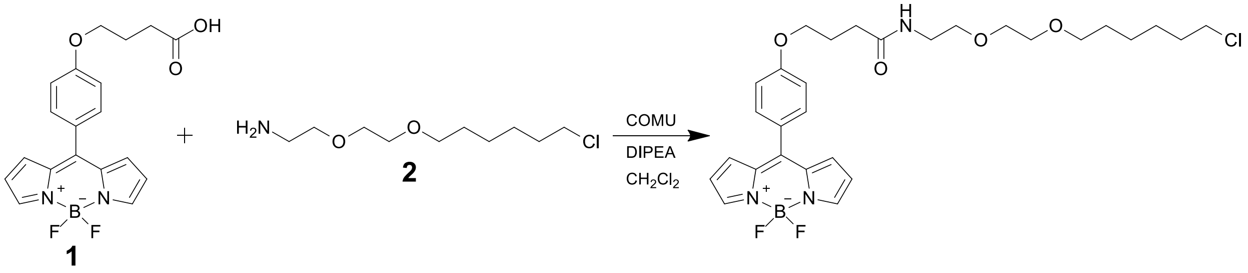

Scheme S1. Synthesis of **BODIPY-HaloLigand**

Note: **BODIPY 1** and 2-(2-((6-Chlorohexyl)oxy)ethoxy)ethanamine (**2**) were prepared according to published procedures. Abbreviations: DIPEA = *N,N*-Diisopropylethylamine; COMU: (1-Cyano-2-ethoxy-2-oxoethylidenaminooxy)dimethylamino-morpholino-carbenium hexafluorophosphate.

BODIPY-HaloLigand.

To a solution of **BODIPY 1** (56 mg, 0.15 mmol) and compound **2** (105 mg, 0.45 mmol) in anhydrous CH_2Cl_2 (5 ml) was added 0.2 ml of DIPEA under argon atmosphere. Then, COMU (70 mg, 0.16 mmol) was added and the reaction mixture was stirred at room temperature for 1 hour. The reaction mixture was diluted with 100 ml of CH_2Cl_2 and the organic phase was washed with 0.1 M HCl (3 x 30 ml), water (1 x 30 ml), 0.1 NaHCO3 (3 x 30 ml), water (1 x 30 ml) and brine (1 x 30 ml), dried over $MgSO_4$, filtered and concentrated under vacuum. Purification by column chromatography on silica gel (eluent: 100 CH_2Cl_2/1 CH_3OH) afforded desired **BODIPY-HaloLigand** as an orange solid. Yield: 43 mg (49%).

^{1}H NMR (300 MHz, $CDCl_3$) δ_H 7.91 (br s, 2H), 7.55 (d, *J* = 8.7 Hz, 2H), 7.05 (d, *J* = 8.7 Hz, 2H), 6.97 (d, *J* = 4.0 Hz, 2H), 6.55 (dd, *J* = 4.0, 1.4 Hz, 2H), 6.07 (br s, 1H), 4.14 (t, *J* = 5.8 Hz, 2H), 3.59 (m, 12H), 2.45 (t, *J* = 6.9 Hz, 2H), 2.23 (q, *J* = 6.1 Hz, 2H), 1.79 (q, *J* = 6.1 Hz, 2H), 1.63 (q, *J* = 5.5 Hz, 2H), 1.45 (m, 4 H); **^{13}C NMR** (75 MHz, $CDCl_3$) δ_C 172.17, 161.58, 147.56, 143.54, 134.98, 132.59, 131.47, 126.49, 118.39, 114.70, 71.41, 70.41, 70.16, 69.95, 67.47, 45.12, 39.38, 32.78, 32.63, 29.59, 26.80, 25.55, 25.16; **^{19}F NMR** (282 MHz, $CDCl_3$) δ_F –145.64 (q, J_{FB} = 28.6 Hz); **HRMS** (ESI-TOF) m/z 598.2429 ($C_{26}H_{37}BF_2N_4O$, $[M+Na]^+$, requires 598.2431).

Figure S1

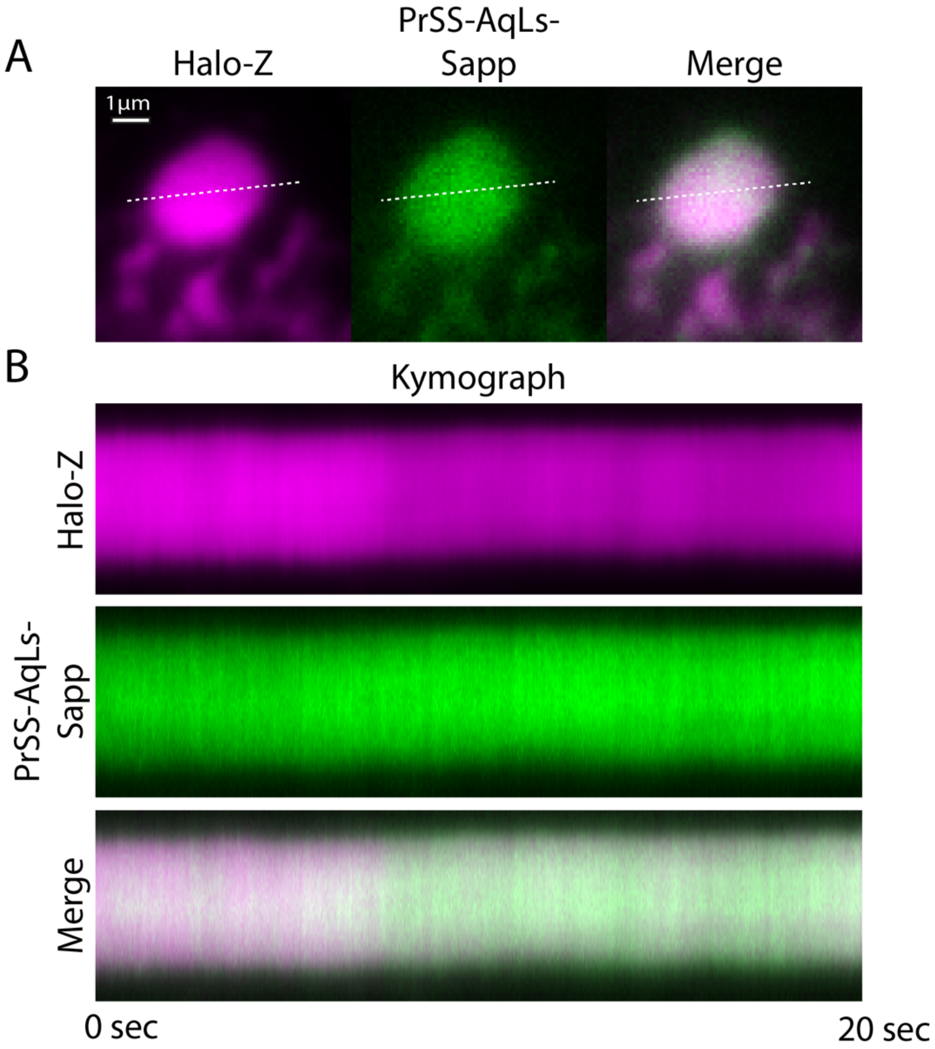

Figure S1.

CHO-K1 cells were transfected with HaloTagged Z-α_1-antitrypsin (Halo-Z) and PrSS-AqLs-sapphire (PrSS-AqLs-Sapp) 48 hours prior to imaging by HILO microscopy. (A) An ER inclusion containing Halo-Z and PrSS-AqLs-Sapp. Dashed line denotes linear ROI used to generate (B) a fluorescence intensity kymograph displaying a 20 second imaging period at 50Hz acquisition rate.

Figure S2

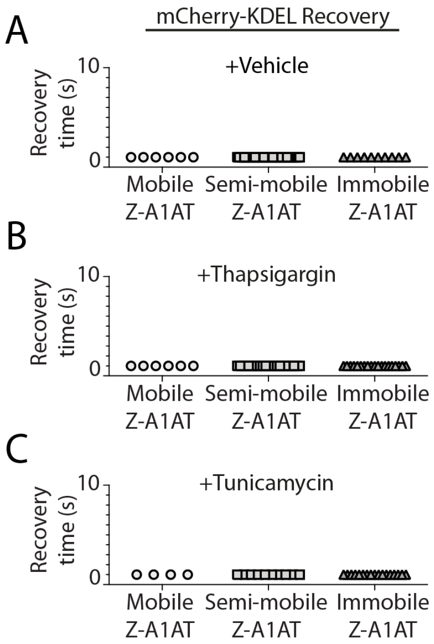

Figure S2.

CHO-K1 cells were transfected with YFP-Z and mCherry-KDEL 48 hours prior to treatment for 8 hours with (A) DMSO (vehicle), (B) 0.02 µM thapsigargin, or (C) 2 µg/ml tunicamycin before imaging. Recovery time of mCherry-KDEL describes the time for ΔI of bleached and control ROIs to homogenise to below 10 %. Number of cells analysed (n) is shown in figure 4A, which displays corresponding YFP-Z mobility phenotypes for this experiment. Data was collected over at least 3 independent experiments.

Figure S3

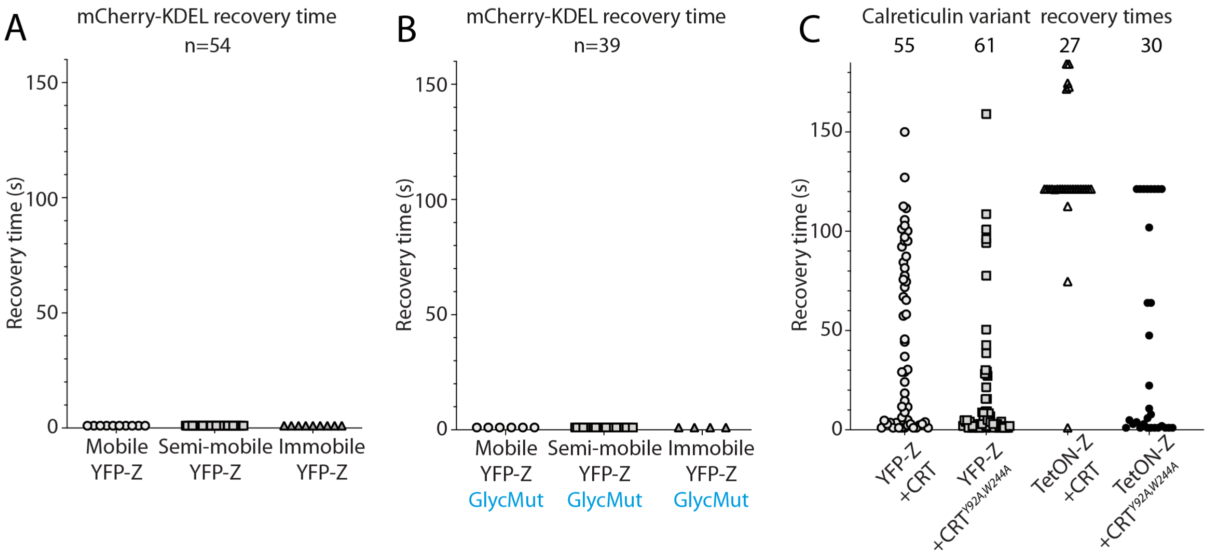

Figure S3.

(A) CHO-K1 cells were transfected with YFP-Z and mCherry-KDEL 48 hours prior to assessment of large ER inclusions by two-colour ID-FRAP. Recovery times of mCherry-KDEL (time for fluorescence homogenisation between control and bleach ROIs within the same inclusion below 10%) are shown, grouped by mobility phenotype of YFP-Z in that inclusion. n denotes number of cells analysed. (B) CHO-K1 cells were transfected with YFP-Z^{N46A,N83A,N247A} (YFP-Z GlycMut) and mCherry-KDEL 48 hours prior to assessment of large ER inclusions by two-colour ID-FRAP, as in A. (C) ID-FRAP recovery times of Halo-CRT and Halo-CRT^{Y92A,W244A} as shown in figure 4D&E, compiled from all groups of YFP-Z mobility, compared with ID-FRAP recovery times of Halo-CRT and Halo-CRT^{Y92A,W244A} transiently transfected into cells with dox-inducible expression of untagged Z-α_1-antitrypsin, induced for 48 hours. Numbers above groups denote number of cells analysed over at least three independent experiments.

Figure S4

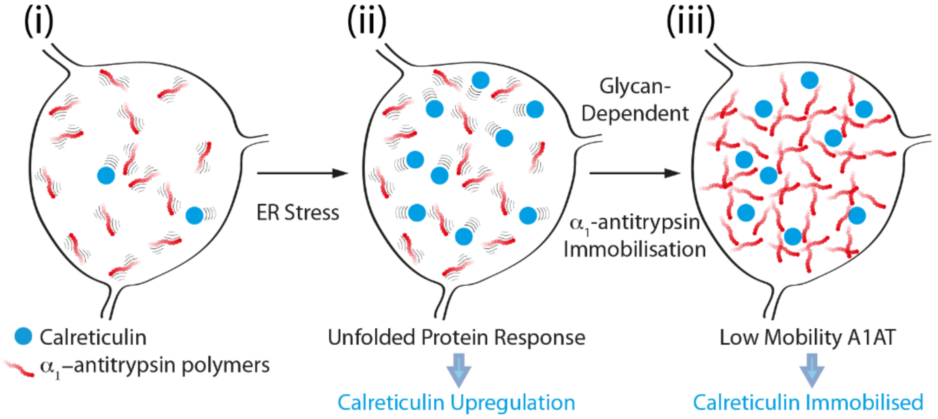

Figure S4.

A cartoon depicting the potential effects of calreticulin upregulation on protein mobility in the ER. (i) Calreticulin (blue circles) and Z-α_1-antitrypsin polymers (linked red circles) move with relative freedom throughout an ER inclusion. (ii) ER stress leads to upregulation of chaperones, including calreticulin, via the unfolded protein response. (iii) Increased calreticulin levels promote Z-α_1-antitrypsin polymer immobilisation by a mechanism that involves Z-α_1-antitrypsin N-linked glycans. Reduced Z-α_1-antitrypsin mobility leads to a reduction in the mobility of calreticulin itself, plausibly through altered polymer arrangement.

Figure S5

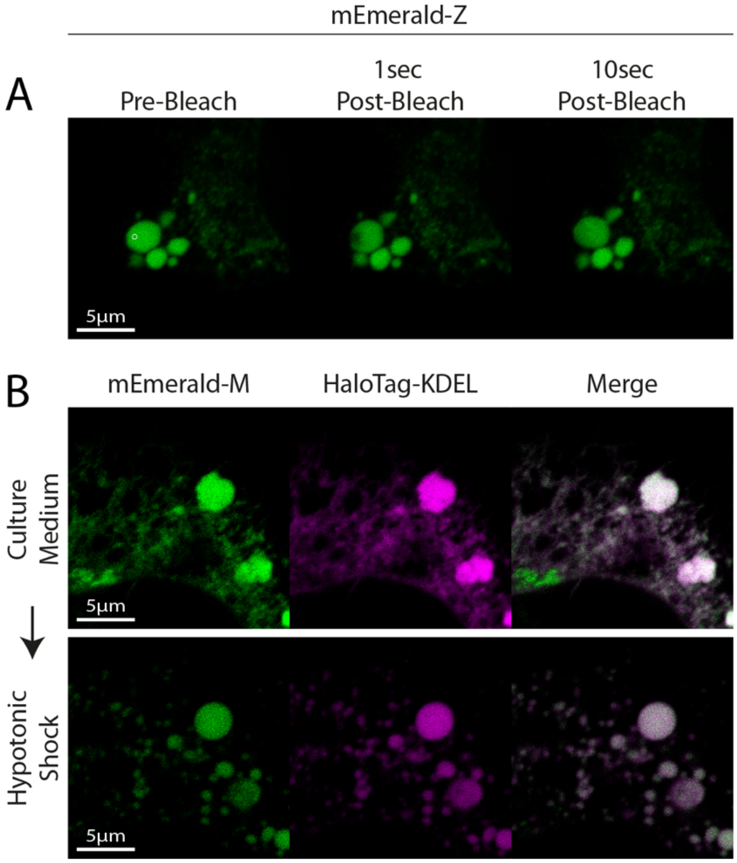

Figure S5.

(A) CHO-K1 cell expressing mEmerald-tagged Z-α_1-antitrypsin and HaloTag-KDEL labelled with TMR HaloTag ligand (not shown) were analysed by ID-FRAP to assign α_1-antitrypsin mobility phenotype. Note bleached region within inclusion recovers by 10 seconds post-bleach. (B) ER inclusions of cells containing “mobile” mEmerald-M, imaged before (upper panels) and after (lower panels) 5 minutes treatment with hypotonic buffer.

Figure S6

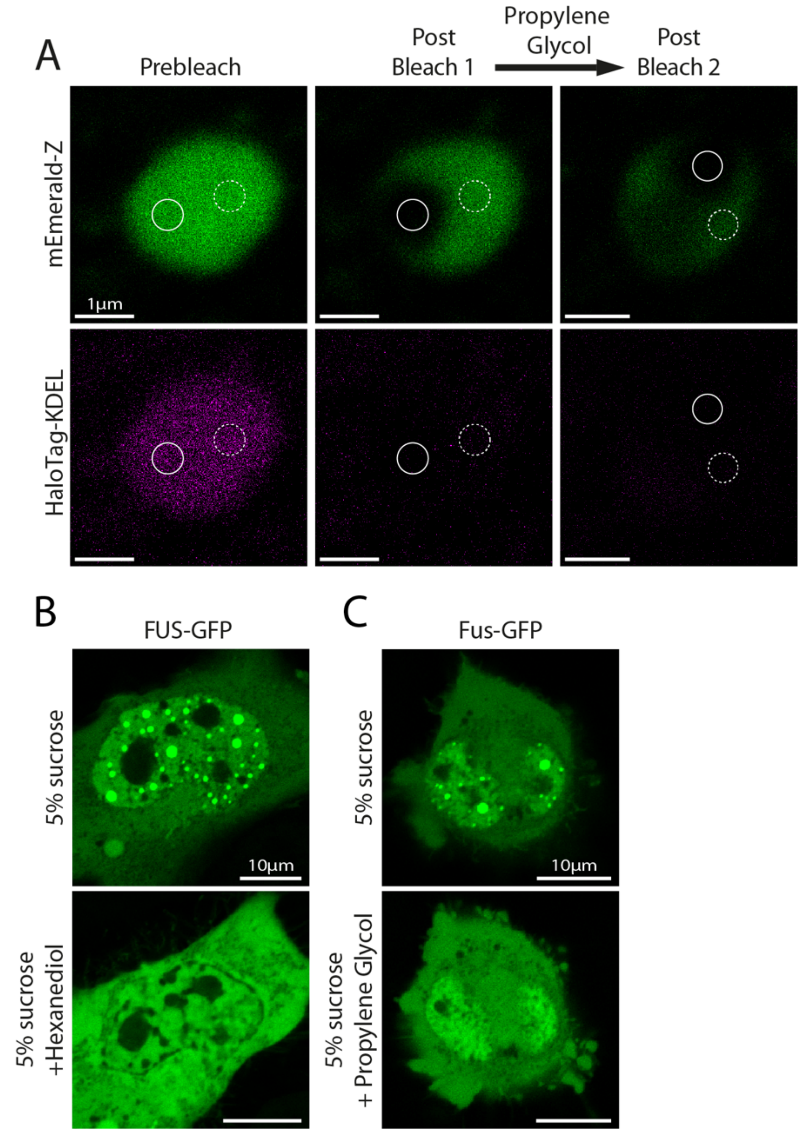

Figure S6.

(A) CHO-K1 cells were transiently transfected with expression plasmids encoding mEmerald-Z and HaloTag-KDEL and were analysed by ID-FRAP. Images show ER inclusions of "immobile" mEmerald-Z before (left panels) and immediately after photo bleach (central panels). The same cell was subsequently treated with 5 % v/v propylene glycol for 20 minutes before FRAP assessment of Z-α_1-antitrypsin mobility in the same inclusion (right panel). (B-C) CHO-K1 cells were transiently transfected with expression plasmids encoding FUS-GFP and were treated with 5 % sucrose for 20 minutes to induce FUS-GFP condensate formation in the nucleus (upper image). Cells were then treated with (B) 4 % w/v 1,6-hexanediol or (C) 5 % v/v propylene glycol prior to imaging (lower image).

Figure S7

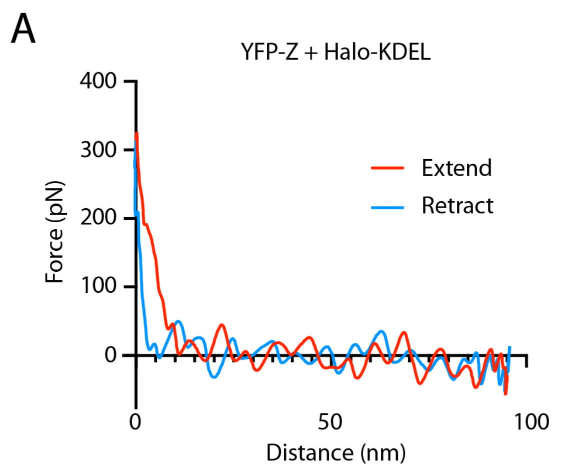

Figure S7.

Example atomic force microscopy force-curves of a YFP-Z punctum extracted from detergent-lysed ER inclusions of CHO cells expressing YFP-Z and (A) Halo-KDEL or (B) Halo-CRT. AFM was performed on glass substrate.

Figure S8

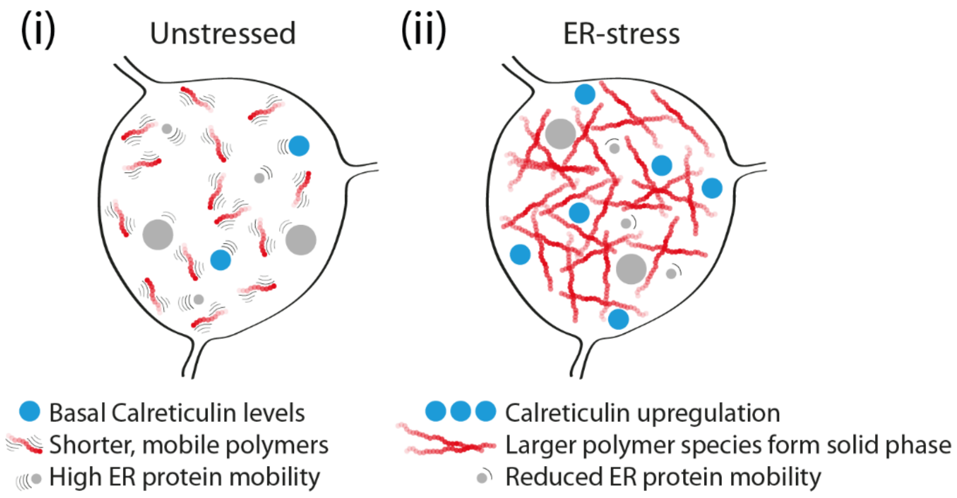

Figure S8.

Cartoon depicting implications of raised calreticulin levels on Z-α_1-antitrypsin physical state - a working model of Z-α_1-antitrypsin immobilisation during ER stress. (i) Calreticulin (blue circles), other ER proteins (grey circles) and Z-α_1-antitrypsin polymers (linked red circles) move with relative freedom throughout an ER inclusion. (ii) ER stress leads to upregulation of chaperones, giving rise to larger polymer-containing species that form a solid matrix within the ER inclusion, reducing the mobility of ER proteins, including calreticulin.

Figure S9

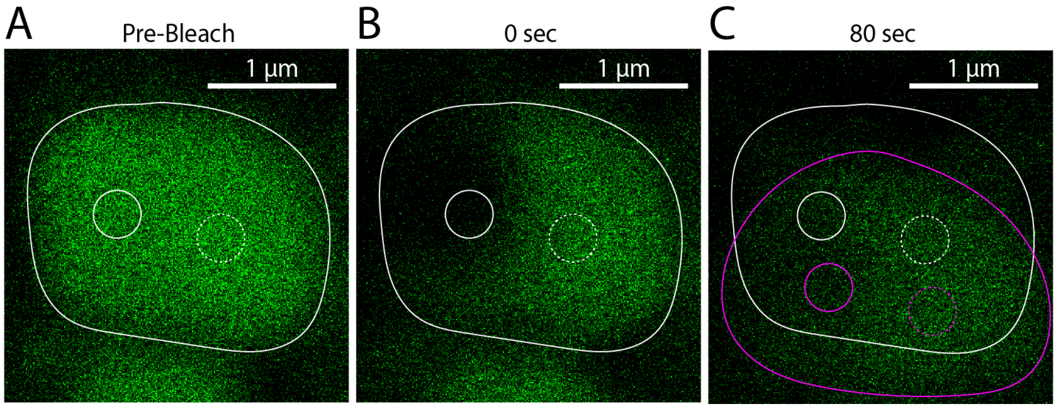

Figure S9.

Example of inclusion movement during imaging. White line circles the perimeter of the inclusions at a pre-bleach time point with corresponding ROI positions in white. Magenta line circles the perimeter of the inclusion 80 seconds post-bleach, with manually adjusted ROI positions relative to the inclusion perimeter shown in magenta. (A) prebleach, (B) immediately post-bleach, and (C) 80 seconds post-bleach frames are shown. ROIs are differentiated as solid-line circles (bleached ROI) and dashed-line circles (control ROI).

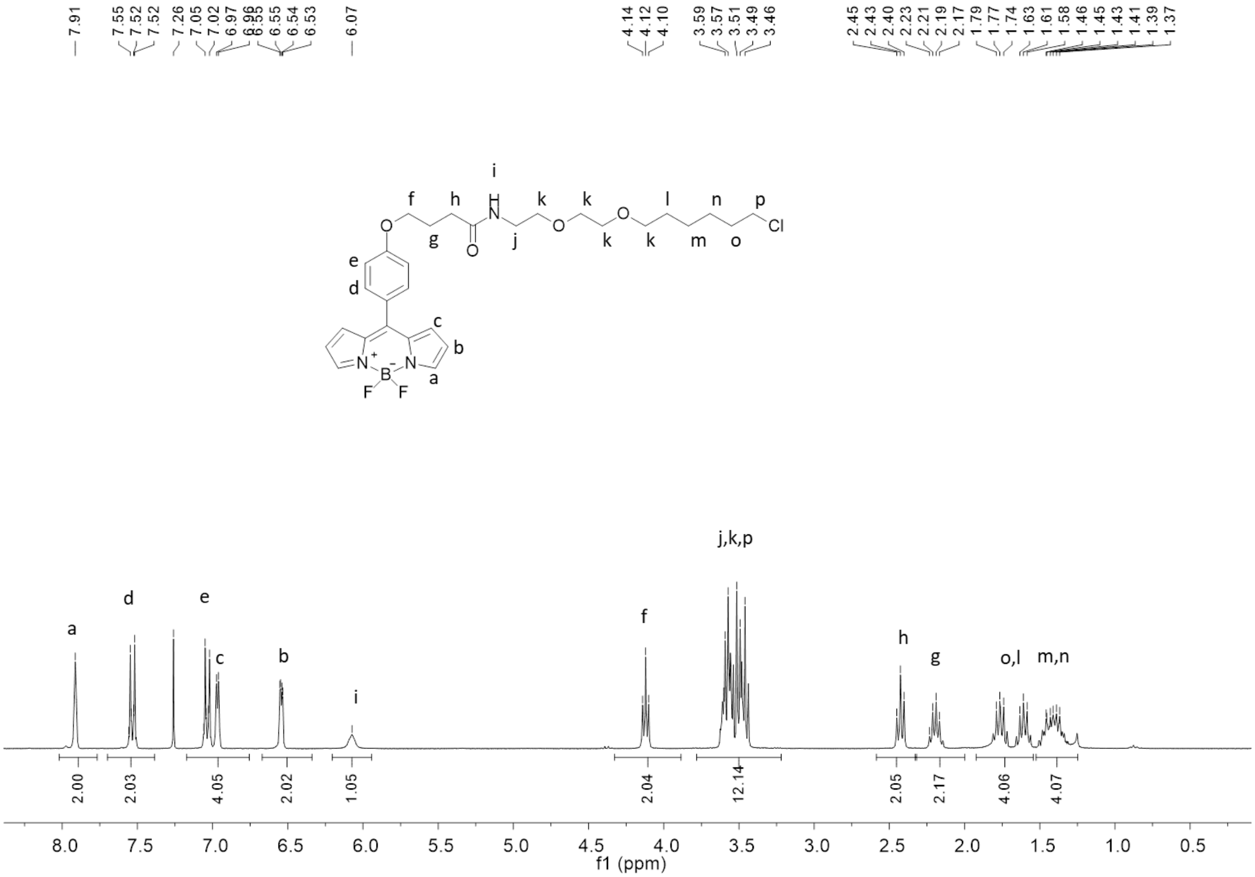

Figure S10.

^{1}H NMR spectrum of **BODIPY-HaloLigand** (300 MHz, $CDCl_3$).

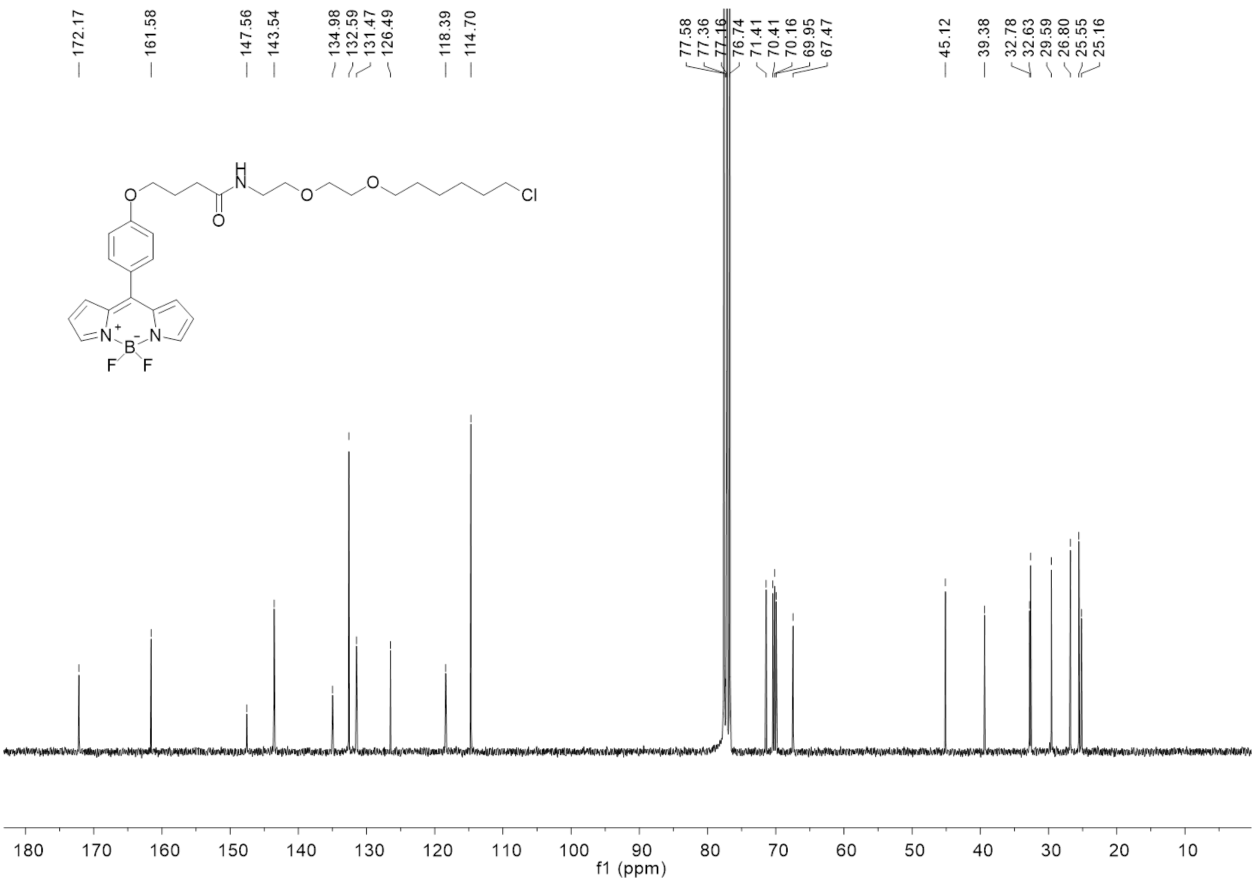

Figure S11.

^{13}C NMR spectrum of **BODIPY-HaloLigand** (75 MHz, $CDCl_3$).

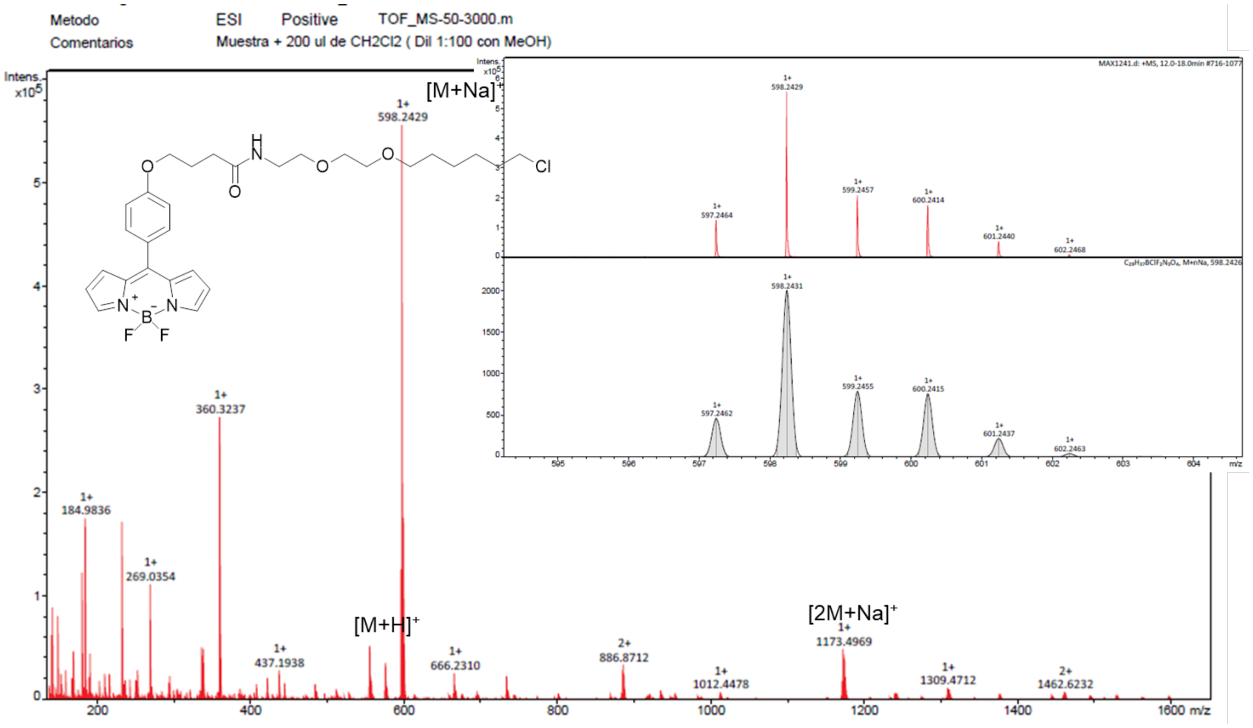

Figure S12.

HR MS (ESI+TOF) mass spectrum of BODIPY-HaloLigand

Movie S1.

HILO microscopy image series of large ER inclusions in CHO-K1 cell expressing (upper panels) HaloTag-M-α_1-antitrypsin (HaloTag-M) (JF646 labelled) or (lower panels) HaloTag-Z-α_1-antitrypsin (HaloTag-Z) (JF646 labelled), together with ER localised AqLs-Sapphire (PrSS-AqLs-Sapp) transfected 48 hours prior to imaging. Note mobile AqLs GEMs in HaloTag-M ER inclusions versus static AqLs GEMs in HaloTag-Z ER inclusions. Scale bar shows 1 um.

Movie S2.

CHO-K1 cell expressing m-Emerald-Z and ER-HaloTag (TMR labelled), transfected 48 hours prior to imaging. Time series shows ER swelling during addition of hypotonic buffer (44 mOsmol/kg) during imaging.

Movie S3.

Single particle tracking of HaloTag-KDEL proteins labelled with PA-JF646 ligand in the ER of a COS7 cell co-transfected with mEmerald-KDEL to visualise underlying ER structure. Image series acquired at 167 Hz.

Movie S4.

Single particle tracking of HaloTag- Z-α_1-antitrypsin proteins labelled with PA-JF646 ligand. COS7 cells were co-transfected with HaloTag- Z-α_1-antitrypsin (HaloTag-Z), and mEmerald-KDEL (ER-Emerald) to visualise underlying ER structure, treated with DMSO (vehicle control) or 0.02 µM thapsigargin (ER Stress (TG)) for 8 hours after PA-JF646 labelling. Image series acquired at 167 Hz.

Movie S5.

Single particle tracking of HaloTag-Z-α_1-antitrypsin proteins in COS7 cells treated with 0.02 µM thapsigargin (data from Movie S4, right panel). Shown is the raw fluorescence intensity image of HaloTag-Z-α_1-antitrypsin proteins labelled with PA-JF646 (top-left), raw HaloTag Z-α_1-antitrypsin fluorescence with assigned tracked particles encircled, with a 500-frame track visible (top-right), full length particle tracks rendered both alone (bottom-left) and overlayed on mEmerald-KDEL fluorescence to show ER structure (bottom-right). HaloTag protein fluorescence is only

designated as a tracked particle (circled in top-right panel) if assigned to a track of 30 or more steps.

Table S1. A list of primers and expression plasmids used in this study.

**Lab stock primer number**	**Primer name**	**Primer sequence**	**Description**
**1474**	EMERALD_GIB-A1AT_1.S	GGCCGTCGCCGTGAGCAAGGGCGAGGAG	Forward primer amplifying mEmerald for replacement of YFP in YFP-Z-A1AT by Gibson assembly
**1475**	EMERALD_GIB-A1AT_2.AS	GCTCGTCCTTCTTGTACAGCTCGTCCATGC	Reverse primer amplifying mEmerald for replacement of YFP in YFP-Z-A1AT by Gibson assembly
**1476**	A1ATBACKBONE_GIB-EM_1.S	GCTGTACAAGAAGGACGAGCTGGCCAGATCTGG	Forward primer amplifying plasmid backbone and A1AT from YFP-Z-A1AT, for receipt of mEmerald by Gibson assembly
**1477**	A1ATBACKBONE_GIB-EM_2.AS	CCTTGCTCACGGCGACGGCCAGGCCGAG	Reverse primer amplifying plasmid backbone and A1AT from YFP-Z-A1AT, for receipt of mEmerald by Gibson assembly
**890**	A1AT_GlycNul_mut_1.S	CCAGCTGGCACACCAGTCCGCCAGCACCAATATCTTCTTCT	Forward primer amplifying glycosylation null A1AT region from synthetic gene fragment by Gibson assembly
**891**	A1AT_GlycNul_mut_2.AS	GGAAGAAGATGGCGGTGGCAGCGCCCAG	Reverse primer amplifying glycosylation null A1AT region from synthetic gene fragment by Gibson assembly
**892**	A1AT_backbone_for_GlycMut_1.S	TGCCACCGCCATCTTCTTCCTGCCTGATGAG	Forward primer amplifying A1AT backbone for glycosylation null A1AT region insertion by Gibson assembly
**893**	A1AT_backbone_for_GlycMut_2.AS	CGGACTGGTGTGCCAGCTGGCGGTATAG	Reverse primer amplifying A1AT backbone for glycosylation null A1AT region insertion by Gibson assembly
**1297**	Calreticulin_Y105A_FWD	GGAAACAGCTTCACAGCGCCGCCCCCACAGTC	Forward primer to introduce Y105A mutation into calreticulin
**1298**	Calreticulin_Y105A_REV	GACTGTGGGGGCGGCGCTGTGAAGCTGTTTCC	Reverse primer to introduce Y105A mutation into calreticulin
**1349**	Calreticulin_W244A_FWD	ATCACTGGGGGTTCCGCCTCTCCGTCCATCTC	Forward primer to introduce W244A mutation into calreticulin
**1350**	Calreticulin_W244A_REV	GAGATGGACGGAGAGGCGGAACCCCCAGTGAT	Reverse primer to introduce W244A mutation into calreticulin
**Lab stock plasmid number**	**Plasmid name**	**Reference**	**Description**
**157**	Halotag_Z AAT	PMID: 27601439	HaloTagged Z-A1AT
**176**	eYFP_M AAT_glyser linker	PMID: 27601439	YFP-tagged M-A1AT
**177**	eYFP_Z AAT_glyser linker	PMID: 27601439	YFP-tagged Z-A1AT
**659**	mEmerald-M-A1AT (glyser linker)	This Paper	mEmerald-tagged M-A1AT
**660**	mEmerald-Z-A1AT (glyser linker)	This Paper	mEmerald-tagged Z-A1AT
**427**	pcDNA3.1_Z AAT	PMID: 27601439	Untagged Z-A1AT
**506**	pcDNA3.1_M AAT	PMID: 27601439	Untagged M-A1AT
**509**	peYFP-C_Z-A1AT_N70A-N107A-N271A	This Paper	YFP-tagged M-A1AT glycosylation-null mutant
**510**	peYFP-C_M-A1AT_N70A-N107A-N271A	This Paper	YFP-tagged Z-A1AT glycosylation-null mutant
**409**	pFLAG_ER-HaloTag	PMID: 29648785	ER localised HaloTag
**190**	pmCherry-KDEL-N3	ClonTech	ER localised mCherry
**656**	pPrSS-mEmerald-KDEL	This Paper	ER localised mEmerald
**632**	HaloTag-Calreticulin	PMID: 30224760	HaloTagged human calreticulin
**666**	HaloTag-Calreticulin-Y92A-W244A	This Paper	HaloTagged human calreticulin functional mutant
**797**	rCRT_GFP	PMID: 16617114	GFP-tagged rat calreticulin
**781**	pLH1337-PrSS-AqLs-HaloTag	This paper	ER localising HaloTagged AqLs GEM
**752**	pLH1337-PrSS-AqLs-Sapphire	This Paper	ER localising Sapphire-tagged AqLs GEM
**919**	pcDNA3.1/hygro(-)-mEmerald-Neuroserpin_G392E	This Paper	mEmerald-tagged G392E mutant of human neuroserpin
**773**	FUS-GFP	PMID: 29677515	GFP-tagged fused in sarcoma (FUS)
